# Population structure and genetic diversity of U.S. wheat varieties

**DOI:** 10.1002/tpg2.20196

**Published:** 2022-03-10

**Authors:** Sajal R. Sthapit, Travis M. Ruff, Marcus A. Hooker, Deven R. See

**Affiliations:** ^1^ Dep. of Plant Pathology Washington State Univ. Pullman WA 99164 USA; ^2^ The Land Institute 2440 E Water Well Rd Salina KS 67401 USA; ^3^ USDA–ARS Wheat Health Genetics, and Quality Research Unit Pullman WA 99164 USA

## Abstract

The United States is a major wheat producer with more than a century of wheat (*Triticum aestivum* L.) research and breeding. Using a panel of 753 historical and modern wheat varieties grown in the United States from the early 1800s to present day, we examined population structure and changes in genetic diversity. We used previously mapped high‐quality single‐nucleotide polymorphism (SNP) markers from the wheat 90K SNP array for genotyping. The wheat varieties had a slight hierarchical population structure based on growth habit and then by kernel color within spring varieties and by kernel hardness within winter varieties, which corresponds with geographical distribution of the varieties. Classifying varieties by market class, which is a combination of habit, hardness, and color, accounted for the greatest amount of variation (13.3%). We did not find evidence of decreased genetic diversity of either spring or winter varieties after the release of the first semidwarf wheat variety in 1961. On the contrary, northern and Pacific spring varieties, hard red spring (HRS), hard white spring (HWS), and soft white winter (SWW) had increases in both SNP and haplotype genetic diversity after 1961. The soft white spring (SWS) and soft red winter (SRW) market classes already had high genetic diversity in varieties before 1961 and showed some evidence of decreased diversity after 1961. Examination of temporal trends in genetic diversity also did not indicate long‐term decline in diversity despite occasional fluctuations.

## Core Ideas

- Population structure of U.S. spring wheat has drastically changed after 1961.
- Winter varieties clustered into hard wheat from the Great Plains and soft wheat from elsewhere.
- Genetic diversity in the U.S. wheat populations has not declined greatly with modern breeding.

**Abbreviations**
AMOVA: analysis of molecular variance
HRS: hard red spring
HWS: hard white spring
PIC: polymorphism information content
pos61: varieties released in and after 1961
pre61: varieties released before 1961
SNP: single‐nucleotide polymorphism
SRW: soft red winter
SSR: simple sequence repeat
SWS: soft white spring
SWW: soft white winter.

## 1 INTRODUCTION

The first wheat (*Triticum aestivum* L.) in the Americas was introduced in the West Indies from Spain in 1494 and then spread to Mexico and the western half of the North and South American continents by the Spanish conquest throughout the 16th century (Ball, 1930; Whitaker, 1929). By the late 18th century, these varieties had reached and adapted to the Pacific coast of the United States. On the eastern coast of the United States, wheat production began in the 17th century and moved westward with European settlements (Dalrymple, 1980). Waves of immigration from different parts of Europe continued to bring landraces to the colonies across the country in the 17th and 18th centuries. Nationwide expansion during 1800–1860 was driven by an increased demand for wheat with advances in milling machinery and greater prosperity of consumers. From 1879 to 1929, production continued to expand into more marginal lands, overcoming climate challenges through changes to management practices and varieties (Olmstead & Rhode, 2011). During 1861–1900, the emphasis remained on collecting and introducing foreign wheat varieties to the U.S. farmers, with progeny selection from breeder's crosses becoming the norm by the early 20th century (Ball, 1930; Dalrymple, 1980). Foreign introductions dominated until the mid‐1930s and domestically bred wheat varieties took over only after 1949 (Reitz, 1979). Today, the United States is a major producer of wheat, with an average annual production of nearly 55 Tg between 2014–2018 (http://www.fao.org/faostat/en/#data). The U.S. wheat production is exceeded only by China, India, and the Russian Federation.

Another landmark in U.S. wheat production was the release of its first semidwarf cultivar, Gaines, in 1961 (Vogel, 1964). Semidwarf varieties enabled wheat plants to take advantage of nitrogen fertilizer without lodging to give bigger yields. In 1964, semidwarf varieties were grown on ∼3% of the U.S. wheat acreages, but by 1974, it had increased to >22% (Dalrymple, 1980). As breeding of wheat has become more advanced, there have been concerns about possible loss of genetic diversity. Drastic reduction in genetic diversity of major crops, including wheat, was observed in the 1960s coinciding with the Green Revolution of semidwarf varieties, but the evidence for long‐term decline remains limited (van de Wouw et al., 2010). However, periodic fluctuation in genetic diversity in multiple studies suggests the need for constant vigilance to broaden the genetic base to maintain or increase genetic diversity (Khoury et al., 2021).

Genetic diversity is essential for breeding programs as a source of novel alleles (Kronstad, 1986). Crossing parents from divergent populations can maximize the variability in the segregating populations for breeders to select on and develop new varieties. Breeding programs may, however, opt to choose the most divergent parents from within an adapted germplasm source for a region in order to maximize variability within the bounds of maintaining adaptation (Barrett & Kidwell, 1998). Therefore, assessment of genetic diversity and population structure is helpful for breeding programs to make strategic planning decisions (Mohammadi & Prasanna, 2003; Zhang et al., 2010). In this study, we describe the population structure of and change in genetic diversity in different U.S. wheat populations.

## 2 METHODS

### 2.1 Wheat variety panel

**FIGURE 1:**
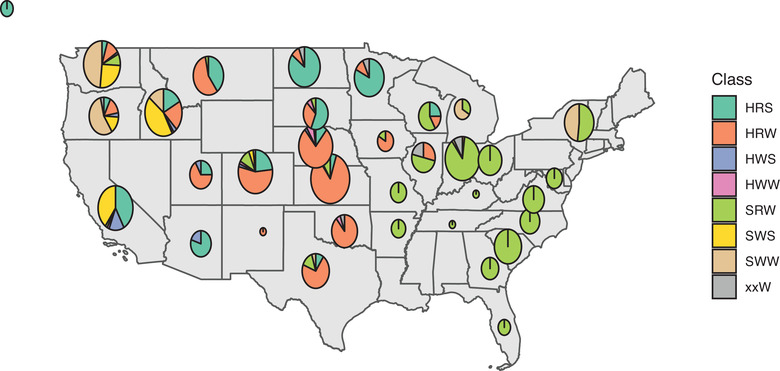
State‐by‐state distribution of 753 U.S. wheat varieties in the study panel color coded by market classes. The four varieties of Alaska are indicated on the top left outside the continental map (not on the same latitude–longitude coordinates). The diameter of the pie chart is proportional to the base 10 logarithm of the number of varieties from the state. HRS, hard red spring; HRW, hard red winter; SRW, soft red winter; SWS, soft white spring; SWW, soft white winter, xxW, winter varieties of unknown market class

We used a panel of 753 U.S. wheat varieties from 1858 to 2014 that included 236 and 517 spring and winter growth habit respectively (Figure 1; Supplemental Table S1). The U.S. National Plant Germplasm System in Aberdeen, ID, provided the seeds. Varieties from the continental United States were assigned to the eastern (Arkansas, Delaware, Florida, Georgia, Iowa, Illinois, Indiana, Kentucky, Louisiana, Maryland, Michigan, Missouri, New York, Ohio, Pennsylvania, South Carolina, Tennessee, Virginia, Vermont, and Wisconsin), the Great Plains (Colorado, Kansas, Nebraska, New Mexico, Oklahoma, and Texas), the northern (Minnesota, Montana, North Dakota, and South Dakota), the Pacific (Arizona and California), and the Pacific Northwest (Idaho, Oregon, Utah, and Washington) wheat producing regions. Four varieties in the panel were from Alaska and not assigned to any regions. The eastern region varieties were 80% soft red winter (SRW), the Great Plains were 76% hard red winter (HRW), the northern were 68% hard red spring (HRS) and 26% HRW, and the Pacific were 48% HRS and 33% SWS. The Pacific Northwest region had a relatively more even distribution of market classes with HRS, HRW, SWS, and SWW comprising 12, 19, 29, and 33% of the varieties respectively. The region assignments are consistent with the production regions defined by the National Association of Wheat Growers (National Association of Wheat Growers, n.d.) with two modifications. First, we assigned Arizona and California into the Pacific region instead of the greater Pacific Northwest because 58 out of 60 varieties from these states were spring and the production region in these states were separated from the Pacific Northwest by the southern half of Oregon. Second, we assigned varieties from Utah to the Pacific Northwest because of the proximity of the Utah and Idaho wheat growing areas.

**Core Ideas**

- Population structure of U.S. spring wheat has drastically changed after 1961.
- Winter varieties clustered into hard wheat from the Great Plains and soft wheat from elsewhere.
- Genetic diversity in the U.S. wheat populations has not declined greatly with modern breeding.

### 2.2 Genotyping

We used the large‐scale DNA extraction protocol described by Faris, et al. (2000) to extract DNA from a single plant per variety. As wheat is self‐pollinated, we assumed each variety to be a homogenous population of homozygous genotypes. While a low level of heterozygosity may exist in a wheat variety, capturing such information would result in a several‐fold increase in genotyping costs.

The panel was genotyped using the Illumina Infinium iSelect 90K single‐nucleotide polymorphism (SNP) array platform (Illumina Inc.) at the USDA–ARS Small Grains Genotyping Center in Fargo, ND. Call frequencies for all markers and varieties were calculated using the default GenTrain algorithm in GenomeStudio v2.0.3 (Illumina Inc.). We removed seven varieties with lower call quality (i.e., threshold of ≥10% of the markers with no calls) and recalculated call frequencies on the remaining 753 varieties. Then, the previously mapped SNPs (Wang et al., 2014) with call frequency ≥90% were filtered to obtain 31,230 SNPs, which were called manually in GenomeStudio's diploid module. We excluded SNPs with more than three distinct clusters or with too many samples in the heterozygote cluster for a dataset of 24,033 markers and 753 varieties. After imputation of missing data with LinkImpute (Money et al., 2015), 15,518 SNPs were polymorphic at minor allele frequency ≥.05. Physical position for 10,502 polymorphic SNPs were obtained by querying marker sequences (downloaded from the Triticeae Toolbox; data/SNPtype‐for‐nucleotide‐conversion‐based‐on‐90K‐manifest.txt) against the Chinese Spring reference genome v1.0 (IWGSC et al., 2018). Duplicate markers with the same physical positions, identical probe sequences, but different marker names were removed for a final dataset of 10,391 SNPs. The AB format calls were converted to nucleotide format calls using 90K SNP array wheat manifest file with conversion rules described at: https://malt.pw.usda.gov/t3/sandbox/wheat/snps.php. The solid spine algorithm in the software Haploview v4.2 (Barrett et al., 2005) was used to define haplotypes based on linkage disequilibrium (with D’ > 0.8) resulting in 1,585 haplotype loci with 6.2 SNPs and 15.7 alleles per locus on average. The A, B, and D genomes had 611, 831, and 143 haplotype loci respectively.

### 2.3 Data analysis

#### 2.3.1 Population structure

Population structure was visually assessed with respect to a priori groups based on growth habit, market class, and region using principal component analysis. Principal components were calculated with the dapc function in the ‘dapc’ package in R (Jombart, 2008). The results were visualized in R (R Core Team, 2018) using various data wrangling and plotting functions from the ‘tidyverse’ package (Wickham et al., 2019).

Hierarchical clustering was conducted using Euclidean distance matrix and ward.D2 agglomeration method with the hclust function in the ‘stats’ package (R Core Team, 2018). Varieties were assigned to different clusters with the cutree function in the ‘stats’ package. A cluster dendrogram was generated using the fviz‐dend function in the ‘factoextra’ package (Kassambara & Mundt, 2017), colormap function in the ‘colormap’ package (Karambelkar, 2016), and barcolor function in the ‘dendextend’ package (Galili, 2015).

Bayesian‐model‐based clustering was applied to the entire panel as well as the spring and winter wheat varieties separately using the program STRUCTURE v2.3.4 (Pritchard et al., 2000). The original STRUCTURE algorithm assumed that markers are not linked (Pritchard, Stephens & Donnelly, 2000). Versions 2.0 and later can handle weakly linked markers after tightly linked markers have been pruned (Falush et al., 2003). We chose a cut‐off of 5‐cM distance between consecutive markers to prune out tightly linked markers to use for our STRUCTURE analysis. To sample the markers, we picked a random marker within the first 5‐cM window of a chromosome. The next marker was sampled from the next window that was at least 5 cM away from the previously selected marker. This sampling procedure gave us 396 loci that were at least 5 cM apart from each other from the 1,585 Haploview haplotype loci dataset. We ran simulations in STRUCTURE with 10,000 burn‐in and 10,000 Markov chain Monte Carlo replications with no prior population assignments for *K* between 1 and 15 with 10 replications. Results were examined using the Δ*K* method (Evanno et al., 2005) as implemented in the Structure Harvester v0.6.94 web application (Earl & vonHoldt, 2012) to choose the most likely number of clusters. The indfile from Structure Harvester corresponding to the most‐supported number of *K* was used as input for the program CLUMMP (Jakobsson & Rosenberg, 2007) to generate consensus membership coefficients (*Q* matrix) that indicate membership probability of each variety to the clusters inferred in STRUCTURE. The FullSearch algorithm in CLUMPP was used to generate consensus *Q* matrix based on all the replications of STRUCTURE outputs. The resulting *Q* matrices were used to generate bar plots in R using functions from the ‘tidyverse’ package (Wickham et al., 2019).

Finally, population structure was examined using analysis of molecular variance (AMOVA) (Excoffier et al., 1992) by running the poppr.amova function in the ‘poppr’ package in R (Kamvar et al., 2015) on the genotyping data of 10,391 polymorphic SNP markers. The subpopulations for AMOVA were defined based on growth habit, kernel hardness, kernel color, market class, region, varieties released before 1961 (pre61), and varieties released in and after 1961 (pos61).

### 2.4 Genetic diversity

To examine changes in genetic diversity, we measured both SNP and haplotype genetic diversity. Single nucleotide polymorphism and haplotype diversity were assessed using gene diversity calculated with the formula, *H_e_ =* 1 − Σ*p_i_* ^2^, where *H_e_* is gene diversity at a locus, and *p_i_* is the frequency of the *i*th allele in the population (Weir, 1996). The formula for polymorphism information content (PIC) given by Botstein, et al. (1980) becomes identical to the formula for gene diversity when applied to codominant markers on inbred lines with no heterozygosity (Serrote et al., 2020). For the rest of the paper, we will use the term PIC instead of gene diversity to avoid potential confusion with genetic diversity. We used 1961, the year of release of the first U.S. semidwarf wheat cultivar Gaines as the cutoff to divide the panel into pre61 and pos61 populations. Polymorphism information content values of pre61 and pos61 varieties were compared using the distribution‐free Wilcoxon rank sum test (also known as the Mann–Whitney U‐test) in R using the function wilcox.test from the ‘stats’ package (R Core Team, 2018). The Holm–Bonferroni correction for multiple hypothesis testing was used to determine statistical significance of differences at α ≤ .05 (Holm, 1979). A sliding window analysis of PIC values with a window size of 30 varieties was used to assess temporal trends in genetic diversity of SNP and haplotype markers. Varieties in a population were ordered by year of release. If multiple varieties were released in the same year, then they were ordered alphabetically by their accession number. In the first step, PIC value was calculated for the oldest 30 varieties. In each subsequent step, the oldest variety in the sliding window was replaced by one new variety and the PIC value for the new sliding window was calculated.

## 3 RESULTS

### 3.1 Population structure

**FIGURE 2:**
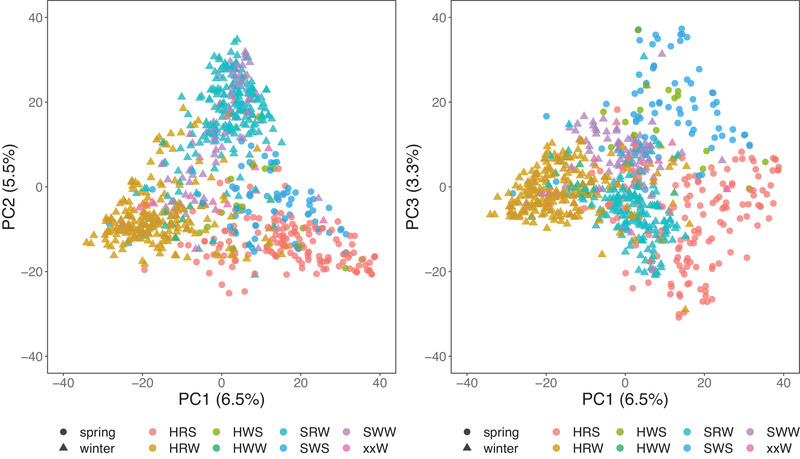
Principal component biplots of U.S. wheat varieties with growth habit denoted by the point shape and market class denoted by color. HRS: hard red spring, HRW: hard red winter, HWS: hard white spring, HWW: hard white winter, SRW: soft red winter, SWS: soft white spring, SWW: soft white winter, xxW: winter varieties of unknown market class

The first three principal components accounted for 15% of the total variation (Figure 2). The first, second, and third principal components separated the varieties in terms of growth habit, kernel hardness, and kernel color, respectively, with considerable overlap in the middle (Supplemental Figure S1). There was one cluster of predominantly spring varieties and two clusters of predominantly winter varieties. The first winter cluster was comprised of SRW, mostly from the eastern region, and SWW from the eastern and Pacific Northwest regions. The second winter cluster comprised primarily of HRW, mostly from the Great Plains and some from the northern and Pacific Northwest regions (Figure 2; Supplemental Figure S2; Supplemental Figure S3). While older spring varieties overlapped with winter varieties, spring varieties after 1960 diverged from winter varieties along the first principal component (Supplemental Figure S4).

**FIGURE 3:**
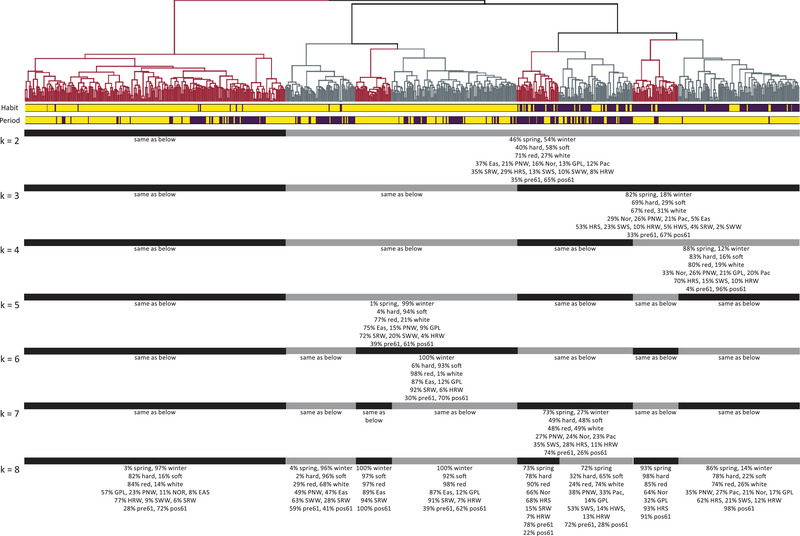
Hierarchical clustering of U.S. wheat varieties using Euclidean distance matrix and Ward.D2 method. In the habit bar, spring and winter varieties are indicated by purple and yellow colors respectively. In the period bar, varieties released pre61 and pos61 are indicated by purple and yellow colors respectively. Eas, Eastern; GPL, Great Plains; HRS, hard red spring; HRW, hard red winter; HWS, hard white spring; Nor, Northern; Pac, Pacific; PNW, Pacific Northwest; pos61, varieties released in and after 1961; pre61, varieties release before 1961; SRW, soft red winter; SWS, soft white spring; SWW, soft white winter

We used an agglomerative algorithm for hierarchical clustering that starts with each variety in its own cluster, which then merge with other similar varieties as the number of clusters are reduced. At *K* = 8, there were four predominantly spring (72–93%) and four predominantly winter (96–100%) clusters (Figure 3). Among the four spring clusters, three were mostly red wheat (74–90%) and one was mostly white wheat (74%). Among the four winter clusters, three were soft wheat (92–97%) and one was hard wheat (82%). At *K* = 7, the two spring wheat clusters with mostly older varieties merged to form a cluster with 74% of the varieties released before 1961. At *K* = 6, the two winter clusters with mostly SRW merged. At *K* = 5, the remaining soft winter wheat cluster (63% SWW and 28% SRW) merged with the SRW cluster. At *K* = 4, two spring clusters with mostly newer varieties merged into a cluster with 96% pos61 varieties and 70% HRS. At *K* = 3, both spring clusters merged into one, while the winter clusters were separated into hard and soft wheat. At *K* = 2, we had one cluster of 97% winter varieties and another cluster with a mix of 46% spring and 54% winter varieties.

**FIGURE 4:**
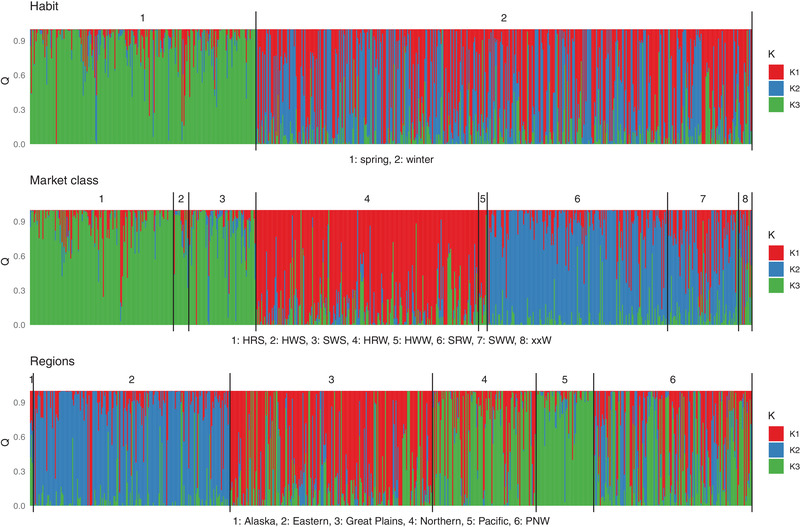
Membership probabilities (*Q*) of U.S. wheat varieties to the three inferred clusters from Bayesian‐model‐based clustering in STRUCTURE using 396 haplotype loci. Varieties have been arranged by growth habit, market class, and regions. HRS, hard red spring; HWS, hard white spring; PNW, Pacific Northwest; SWS, soft white spring; HRW, hard red winter; SRW, soft red winter; SWW, soft white winter; xxW, winter varieties of unknown market class

Bayesian‐model‐based clustering in STRUCTURE applied to the entire panel inferred three clusters based on the Δ*K* plot in Structure Harvester. Winter varieties were inferred into two clusters (K1 and K2) and the spring varieties were inferred into the K3 cluster (Figure 4). Hard red winter and HWW were mostly represented in the K1 cluster, while SRW and, to a slightly lesser extent, SWW were in the K2 cluster. Clusters were also correlated to a slightly lesser extent with geographic regions. As growth habit influences cultivation practices, breeding, and regional adaptation for wheat varieties, we conducted further STRUCTURE analyses within spring and winter varieties separately. The Δ*K* plot of spring varieties inferred three clusters. The HRS varieties were mostly in the K1 and to some extent in the K3 spring clusters. The soft spring (HWS and SWS) varieties were mostly in K2 followed by K3 (Supplemental Figure S5). The Δ*K* plot of winter varieties showed support for two clusters. The soft winter (SRW and SWW) varieties more prevalent in K1 winter cluster and the hard winter (HRW and HWW) more prevalent in the K2 winter cluster (Supplemental Figure S5).

We conducted AMOVA by grouping the panel of varieties based on a priori categories of growth habit, kernel hardness, kernel color, market class, region, and breeding period (pre61 and pos61). Market classes accounted for the greatest amount of variation (13.3%, df = 6) followed by growth habit (8.8%, df = 1), region (8.0%, df = 4), kernel hardness (6.5%, df = 1), kernel color (4.9%, df = 1), and breeding periods (2.5%, df = 1) (Supplemental Table S2). Among the spring varieties, kernel color (10.8%, df = 1) and market classes (10.6%, df = 2) accounted for the most variation followed by kernel hardness (9.6%, df = 1), breeding periods (8.7%, df = 1), and regions (7.4%, df = 4) (Supplemental Table S2). Among winter varieties, market classes (11.3%, df = 3) and kernel hardness (10.8%, df = 1) accounted for the most variation followed by region (8.6%, df = 4), kernel color (5.6%, df = 1), and breeding periods (2.2%, df = 1). Varieties pre61 and pos61 were more differentiated among spring varieties.

Within the eastern and the Great Plains regions, growth habit accounted for the most variation (18% each) followed by market class (11 and 16%. respectively) (Supplemental Table S3). In the other three regions, market class accounted for the most variation ranging from 14% each in northern and Pacific Northwest regions and 22% in the Pacific region. States account for little variation within most regions with a maximum of 10% variation accounted for in the eastern region with varieties from 22 states. Finally, the breeding period of pre61 and pos61 varieties accounted for a great degree of variation (20%) only in the Pacific region varieties.

When ANOVA was conducted within major market classes, effect of breeding period was relatively stronger in spring classes, accounting for 12 and 10% of the variation in HRS and SWS, respectively (Supplemental Table S4). Regional differentiation within market classes were relatively stronger in SWW, HRS, and SWS, accounting for 13, 9, and 9% of the variation, respectively.

### 3.2 Genetic diversity

The entire U.S. panel had median PIC values of .396 and .673 for SNP and haplotype loci, respectively (Supplemental Table S5). Spring varieties had greater diversity than winter varieties. The Pacific region varieties had the highest median PIC for SNPs (.406) and the northern region had the highest median PIC for haplotypes (.651). The Great Plains, despite having the most varieties in the panel, had the lowest SNP and haplotype diversity. Hard red spring and SRW were the most diverse spring and winter market classes, respectively. While HRS had a greater median PIC for SNPs than SRW, their median haplotype PICs were the same.

Polymorphism information content values assessed using SNPs had a highly skewed distribution indicated by large standard deviations with respect to the means (Supplemental Table S5) and wider bulges in violin plots toward the extreme values away from the mean and median, while PIC values assessed using haplotype loci had slightly skewed distribution (Supplemental Figure S6). Distribution‐free nonparametric Wilcoxon rank sum test was used to compare PIC values between pre61 and pos61 varieties at α ≤ .05 with Holm–Bonferroni correction for multiple hypothesis testing. Of the 16 null hypotheses of no change in PIC values before and after 1961, SNP and haplotype dataset rejected 12 and seven, respectively (Table 1). Of the 19 comparisons showing significant changes, 16 showed increase and three showed decreases in genetic diversity. Genetic diversity of SNPs decreased in SRW varieties pos61, while genetic diversity of haplotype loci decreased in northern winter varieties and SWS varieties pos61. Statistically significant increase in both SNP and haplotype genetic diversity was found in Northern spring, Pacific spring, HRS, HWS, and SWW varieties. Genetic diversity in SNP datasets increased in spring varieties, which contributes to the small increase in overall genetic diversity. The greatest increase in SNP marker diversity was in the D genome, but there was no corresponding increase seen when using haplotype marker diversity (Supplemental Figure S6). Genetic diversity of winter varieties did not change much between pre61 and pos61 varieties.

**TABLE 1:** Polymorphism information content (PIC) of U.S. wheat subpopulations before and since 1961

Population	No.	Single‐nucleotide polymorphism					Haplotype				
		Mean	Median	*P*	Rank	α_HB_	Mean	Median	*P*	Rank	α_HB_
All USA ≤1960	249	.339	.378	.000*	1	.002	.636	.664	.096	25	.006
≥1961	504	.364	.399	–	–	–	.651	.671	–	–	–
**Spring by region**											
All USA≤1960	70	.335	.382	.000*	2	.002	.612	.642	.185	26	.007
≥1961	166	.369	.411	–	–	–	.622	.653	–	–	–
Northern ≤1960	19	.269	.266	.000*	3	.002	.502	.532	.000*	9	.002
≥1961	54	.338	.384	–	–	–	.584	.617	–	–	–
Pacific ≤1960	23	.280	.340	.000*	4	.002	.513	.552	.000*	16	.003
≥1961	35	.335	.382	–	–	–	.551	.586	–	–	–
PNW ≤1960	19	.316	.388	.000*	5	.002	.585	.620	.242	28	.010
≥1961	53	.349	.389	–	–	–	.597	.629	–	–	–
**Winter by region**											
All USA ≤1960	179	.324	.371	.019	23	.005	.618	.650	.927	32	.05
≥1961	338	.331	.371	–	–	–	.621	.644	–	–	–
Eastern ≤1960	86	.318	.357	.005	21	.004	.606	.641	.094	24	.006
≥1961	116	.310	.348	–	–	–	.593	.635	–	–	–
Great Plains ≤1960	53	.289	.306	.000*	18	.003	.567	.600	.348	30	.017
≥1961	132	.298	.307	–	–	–	.563	.591	–	–	–
Northern ≤1960	12	.283	.278	.233	27	.008	.547	.569	.001*	19	.004
≥1961	23	.275	.287	–	–	–	.524	.556	–	–	–
PNW ≤1960	28	.285	.337	.000*	11	.002	.550	.594	.004	20	.004
≥1961	65	.308	.355	–	–	–	.577	.604	–	–	–
**Market classes**											
HRS ≤1960	27	.296	.302	.000*	6	.002	.551	.587	.000*	12	.002
≥1961	123	.357	.400	–	–	–	.603	.637	–	–	–
HWS ≤1960	9	.239	.198	.000*	7	.002	.446	.494	.000*	10	.002
≥1961	7	.324	.408	–	–	–	.542	.571	–	–	–
SWS ≤1960	34	.311	.360	.283	29	.013	.574	.616	.000*	14	.003
≥1961	36	.311	.346	–	–	–	.538	.573	–	–	–
HRW ≤1960	65	.282	.301	.000*	15	.003	.558	.587	.799	31	.025
≥1961	167	.294	.302	–	–	–	.561	.585	–	–	–
SRW ≤1960	83	.316	.353	.000*	17	.003	.604	.638	.008	22	.005
≥1961	105	.306	.342	–	–	–	.585	.625			
SWW ≤1960	24	.265	.278	.000*	8	.002	.514	.542	.000*	13	.003
≥1961	50	.303	.343	–	–	–	.567	.602			

*Note*. HRS, hard red spring; HWS, hard white spring; SWS, soft white spring; HRW, hard red winter; SRW, soft red winter; SWW, soft white winter.

* Indicates *P* values significant after applying the Holm‐Bonferroni correction for multiple hypotheses testing. The Holm‐Bonferroni correction is conducted by ranking the hypotheses by their *P* values from smallest to largest. For each hypothesis, a rank‐specific significance threshold is calculated with the formula α_HB_ = α/(*n* ‐ *r* +), where α is the overall alpha level (.05), *n* is number of hypothesis (32), and *r* is the rank of the hypothesis. A hypothesis is considered significant if its *P* value is less than its rank‐specific α_HB_. *P* values of .000 indicate values <.001.

Temporal trends in genetic diversity using 30‐variety sliding windows showed only minor fluctuations in genetic diversity in both SNPs (Supplemental Figure S7) and haplotypes (Supplemental Figure S8) in most populations. There was a period of lowered diversity in spring varieties from 1918 to 1964 (in sliding windows 14–48). The 10 earliest HRS sliding windows also had slightly lower genetic diversity. The most recent dip in diversity was in winter varieties in the sliding windows 476–480 covering the period between 1999 and 2007. However, none of these dips remained persistent over the long‐term suggesting overall stability of genetic diversity in the U.S. wheat varieties.

As haplotype markers are multiallelic, we examined changes in haplotype alleles per locus and proportion of lost and new alleles with respect to the pre‐1930 varieties in our panel. We found that 25–39% of alleles were lost and 13–35% alleles were new in every decade indicating the dynamic nature of genetic changes in wheat. Alleles per locus appeared to be highest in the varieties from before 1930 and from the decades between 1970 and 2000 (Supplemental Table S6). Varieties released after 2000 had the highest percentage of lost alleles and less than half of these lost alleles were replaced by new alleles (Supplemental Table S6). While, this may seem like an alarming loss, we found haplotype alleles per locus to be correlated with population size (Supplemental Figure S9). When we examined alleles per locus between equal group sizes, alleles per locus and the proportion of lost and new alleles were relatively more consistent over time (Supplemental Table S6).

## 4 DISCUSSION

### 4.1 Population structure

We assessed the population structure of the U.S. wheat varieties using AMOVA and multiple clustering methods. Analysis of molecular variance on the entire panel showed that market class explained the greatest amount of variation (13.3%, df = 6). Market class also explained ∼11% of variation within the spring and winter populations. More than 85% of the variation was found within populations, which indicates a weak overall population structure in the U.S. wheat variety panel in our study. Previous studies in U.S. wheat have also apportioned 80–90% of the molecular variation within populations rather than between populations. For example, geographic origin, growth habit, and market class accounted for 10–13% (Bonman et al., 2015), 10% (Chao et al., 2010), and 17% (Chao et al., 2007) of the variation between populations, respectively. One notable exception was an older study of 54 Pacific Northwest varieties genotyped with 16 amplified fragment length polymorphism markers that apportioned 30% of the variation to growth habit (Barrett & Kidwell, 1998). Wheat breeding is notable in terms of sharing of breeding lines with common ancestry between national and international breeding programs (Cavanagh et al., 2013), which may account for limited differentiation between populations.

Principal component analysis and Bayesian‐model‐based clustering in STRUCTURE both indicated support for one mostly spring and two mostly winter clusters. The winter clusters comprised of hard winter (HRW and HWW) and soft winter (SRW and SWW) varieties. Hierarchical clustering at *K* = 3 concurred with the clusters of mostly spring varieties, hard winter varieties, and soft winter varieties. As market class is a combination of growth habit, kernel color, and kernel hardness, the AMOVA results are consistent with the hierarchical population structure indicated by the clustering methods.

Older spring and winter varieties clustered closer together in the principal component analysis biplots, while the newer spring varieties diverged away from the rest (Supplemental Figure S4). Grouping varieties by breeding period (pre61 and pos61) accounted for 9% of variation in spring wheat but only 2% in winter wheat in AMOVA, suggesting that newer spring varieties in the United States have diverged more from their predecessors. The two spring market classes (HRS and SWS) also showed relatively higher differentiation (10–12% of the variation) between pre61 and pos61 populations (Supplemental Table S4). Within the Pacific region, grouping by breeding period accounted for 20% of the variation (Supplemental Table S3). This stark change in population structure after 1961 likely is due to increased use of CIMMYT germplasm in the second half of the 20th century in breeding programs of the western United States, especially California (Balfourier et al., 2019).

Previous studies have reported population structure in wheat in terms of geographical origin, growth habit, and improvement status. Balfourier et al. (2019) found geographical origin as the main factor of population structure in a global panel of 632 wheat landrace accessions. Likewise, Bonman et al. (2015) found population to be structured along geographical origin in a panel of over 3,200 accessions with 55% landraces or uncertain status and 45% improved lines. Muleta et al. (2017) studied a panel of over 1,000 spring wheat accessions from 91 countries (56% landraces) and reported population structured by geographic origin with Asian accessions clustering separately from European, North American, and South American accessions. However, in a study panel with nearly 3,000 accessions—but only 5% of them landraces—the population was found to be structured by growth habit, with the landraces mostly occupying the space between spring and winter clusters (Cavanagh et al., 2013). As the oldest varieties in our panel were line selections made from landraces, we also observed the pattern of the older spring and winter varieties clustering together in the middle of the biplot while the newer varieties diverged from the center (Supplemental Figure S4). Likewise, Chao et al. (2010) found that growth habit accounted for 10% of the variation in their study of 478 elite U.S. wheat lines and no landraces. In our panel, when habit or market classes were nested within regions, they tended to explain slightly more variation (Supplemental TableS3) than when regions were nested within market classes (Supplemental Table S4). Therefore, it appears that adaptation to geographic region first influences the population structure in the early stages of crop improvement when landraces are being adapted. As breeding programs mature, greater specialization within growth habit and market classes follows with adapted elite by elite crosses being used for most variety development.

### 4.2 Genetic diversity

Studies of genetic diversity often report mean PIC values as a measure of genetic diversity. Because of the skewed distribution of PIC values, we report median in addition to mean PIC values (Supplemental Table S5; Table 1). The mean PIC values for SNPs in our study panel (.361, .375, and .332 for all, spring, and winter varieties, respectively) are higher than previously reported from a low of .160 in elite U.S. and Mexican varieties (Chao et al., 2010) to a high of .330 in synthetic hexaploid lines (Bhatta et al., 2018). However, PIC values in different studies cannot be directly compared unless the same markers are genotyped. Our finding that spring varieties had greater PIC values than winter varieties was consistent with previous studies (Bonman et al., 2015). Hard red spring and SRW were the most diverse spring and winter market classes respectively, which is consistent with previous findings based on simple sequence repeat (SSR) markers, albeit on a smaller panel size (Chao et al., 2007).

White et al. (2008) reported an overall increasing trend in U.S. wheat genetic diversity starting in the 1950s. Their U.S. variety panel included 96 varieties (48 of which are in the current study) covering a period from 1840 to 2002. A recent study of 142 spring and 178 winter wheat varieties of the Pacific Northwest also showed support for increase in genetic diversity of spring wheat from the 1970s but limited change in winter wheat diversity (Sthapit et al., 2020). Consistent with these studies of U.S. wheat, we did not find evidence of decline in genetic diversity of varieties released after 1960. The plot of PIC values by sliding window indicated a relative dip in genetic diversity of winter varieties from 1999 and 2007 (Supplemental Figure S7; Supplemental Figure S8). Each of these sliding windows had 20 or more HRW varieties, which may explain the temporary dip that was alleviated in subsequent windows with the replacement of HRW by SWW varieties. Overall, the remarkably stable trend in 30‐variety sliding windows also supports the conclusion that there have not been drastic losses in the quantitative level of genetic diversity in U.S. wheat populations.

Significant changes in genetic diversity between pre61 and pos61 varieties were more common in SNP loci than haplotype loci, but more instances of significant loss were reported by haplotype loci than by SNPs. Median SNP genetic diversity was lower in the pos61 populations in three out of 16 comparisons, but the difference was significant in only one comparison. Median haplotype genetic diversity was lower in seven out of 16 comparisons and the difference significant in two comparisons. Therefore, haplotype data reported changes in genetic diversity in both directions. A similar pattern of reduced haplotype loci diversity has been reported in modern Australian varieties (Joukhadar et al., 2017). Voss‐Fels et al. (2019) have attributed the observed decrease in haplotype diversity in modern European varieties to cumulative elimination of neutral or deleterious linkage blocks over several decades of breeding rather than a result of loss of useful genetic diversity.

Although SNP markers have become the norm for conducting genetic studies in the past two decades, ascertainment bias resulting from the use of few accessions for SNP discovery may be a concern. Ascertainment bias may be corrected by haplotyping (Balfourier et al., 2019; Lachance & Tishkoff, 2013). Genome‐wide association studies may have greater power to detect marker–trait associations in wheat when using haplotype markers instead of SNPs (Hao et al., 2017). Older studies that have used multiallelic markers like SSRs also reported alleles per locus in addition to PIC as a measure of genetic diversity. Fu and Somers (2009) used SSR alleles per locus to report a net loss of alleles in the newest 20 Canadian HRS varieties compared with the oldest 20 varieties from before 1930. Simple sequence repeats can evolve by replication slippage in addition to point mutation and recombination and hence are hypervariable (Schlötterer, 2000). The haplotypes constructed in our study are a combination of several SNP loci. The fact that SNPs do not evolve by replication slippage may explain why haplotype alleles per locus in our study were highly correlated with number of varieties.

We found evidence of increase in genetic diversity of both SNP and haplotype loci for HRS, HWS, SWW, northern spring varieties, and Pacific spring varieties in the pos61 populations. The HRS market class had the most impressive increase in genetic diversity in our panel, which contrasts with the decrease in genetic diversity of Canadian HRS (Fu & Somers, 2009). The pre61 HRS varieties were mostly from the northern region states of North and South Dakota and Minnesota, while the pos61 population included a lot more varieties developed in the Great Plains, the Pacific, and the Pacific Northwest in addition to the northern states that contributed to increased diversity. Likewise, the pos61 northern spring varieties (all HRS in our panel) had a more even representation of varieties developed in Minnesota, Montana, North Dakota, and South Dakota. The pre61 group was dominated by North Dakota varieties. The pre61 Pacific spring varieties in our panel were all HWS and SWS, but the pos61 population were mostly HRS, which increased the genetic diversity of Pacific varieties. The pre61 HWS had a rather low genetic diversity, as four of the nine varieties were derived from ‘Baart’. Therefore, a reduced reliance on Baart managed to greatly increase the genetic diversity of HWS after 1960. The pre61 SWW population had multiple varieties derived from ‘Arcadian’, ‘Goldcoin’, ‘Hybrid 128’, and ‘Mediterranean’, while the pos61 population had more diverse pedigrees. National and international sharing of germplasm, and especially the influence of CIMMYT lines to develop new varieties, may have helped increase the diversity of spring varieties (Balfourier et al., 2019).

Soft white spring and SRW were the two market classes that had a decrease in genetic diversity of varieties since 1961. For SWS, the decrease was significant only for haplotype loci, while for SRW, the decrease was significant only for SNP loci. The SWS and SRW market classes had the highest or the second highest median PIC values among market classes for both SNP and haplotype loci in the pre61 varieties. Considering their high pre61 genetic diversity, the decline in pos61 may not be alarming. The median PIC of pos61 SRW varieties is still higher than that of other winter market classes. Finally, the northern winter subpopulation had a significant decline in haplotype diversity but a nonsignificant increase in its median PIC of SNP loci (Table 1). The pre61 northern winter population only had 12 varieties (seven HRW, two SRW, and three unknowns), while the pos61 population had 23 varieties, but 21 of them were HRW. The pre61 northern winter population only had 12 varieties: six from Montana, four from Minnesota, and one each from North and South Dakota. In contrast, the pos61 population had 23 varieties, with 12 from Montana, six from South Dakota, four from North Dakota, and only one from Minnesota. Pedigree analysis—visualized in the software Helium (Shaw et al., 2014)—of these 35 northern winter varieties indicates much greater influence of the HRW ‘Cheyenne’ in the pos61 varieties as well as inclusion of closely related cultivars such as ‘Rampart’, ‘Redwin’, ‘Tiber’, and ‘Vanguard’ (Supplemental Figure S10).

## 5 CONCLUSIONS

The objectives of the study were to assess the population structure and change in genetic diversity in the U.S. wheat varieties. We found hierarchical population structure of growth habit and market class with evidence of modern spring varieties diverging the most from older spring and winter varieties. Evidence from the current and previous studies suggest that population structure is first influenced by regional adaptation followed by further specialization within growth habit and market classes. The population structure insights from cluster analyses and AMOVA will be useful for breeding programs to identify adapted parents that are still divergent enough to maximize variability from a cross with reduced risk of losing adaptedness. We did not find evidence of large decline in genetic diversity of wheat varieties after 1960. We also did not find any long‐term trend of genetic diversity loss despite temporary fluctuations based on sliding window analysis. The shift in population structure and increase in genetic diversity of spring varieties is likely a result of greater exchange of germplasm and incorporation of CIMMYT germplasm in development of new varieties after 1960.

## AUTHOR CONTRIBUTIONS

Sajal R. Sthapit: Conceptualization; Data curation; Formal analysis; Investigation; Methodology; Project administration; Validation; Visualization; Writing – original draft; Writing – review & editing. Travis M. Ruff: Methodology; Project administration; Writing – review & editing. Marcus A. Hooker: Methodology; Writing – review & editing. Deven R. See: Conceptualization; Funding acquisition; Resources; Software; Supervision; Writing – review & editing.

## CONFLICT OF INTEREST

The authors declare no conflict of interest.

## Supporting information

Supplemental Materials
The following supplemental materials are available in a separate file.
Supplemental Figure S1. Principal component biplots of U.S. wheat varieties coded by growth habit, kernel hardness, and kernel color.
Supplemental Figure S2. Principal component biplots of U.S. wheat varieties by market classes.
Supplemental Figure S3. Principal component biplots of U.S. wheat varieties by regions. Growth habit is denoted by the point shape and market class is denoted by color. HRS: hard red spring, HRW: hard red winter, HWS: hard white spring, HWW: hard white winter, SRW: soft red winter, SWS: soft white spring, SWW: soft white winter, xxW: winter varieties of unknown market class.
Supplemental Figure S4. Principal component biplots of U.S. wheat varieties from different time periods.
Supplemental Figure S5. Population structure of (a) spring and (b) winter varieties using 396 haplotype loci with varieties grouped by market class and region. Each vertical line represents a variety with the color representing its membership coefficient to the inferred clusters in STRUCTURE. ΔK peaked at 3 clusters for spring and 2 clusters for winter varieties. HRS: hard red spring, HWS: hard white spring, SWS: soft white spring, HRW: hard red winter, SRW: soft red winter, SWW: soft white winter, xxW: winter varieties of unknown market class.
Supplemental Figure S6. Violin plots, box plots, and mean (red dot) values of PIC distribution of U.S. wheat varieties using (a) SNP and (b) haplotype datasets. All SNPs used for this analysis had minor allele frequency ≥ .05 on the entire panel. Details about the datasets used are provided in the methods section.
Supplemental Figure S7. Temporal trends in the polymorphism information content (PIC) of 10,391 SNP loci in (a) both spring and winter varieties, (b) spring varieties, (c) winter varieties, and (d) market classes. The red and blue dots are the mean and median PIC values for the 30‐variety sliding windows.
Supplemental Figure S8. Temporal trends in the polymorphism information content (PIC) of 1585 haplotype loci in (a) both spring and winter varieties, (b) spring varieties, (c) winter varieties, and (d) market classes. The red and blue dots are the mean and median PIC values for the 30‐variety sliding windows.
Supplemental Figure S9. Means (red dot), box plots and violin plots (gray) of haplotype alleles per locus based on subpopulation size. Subpopulations of 20 to 500 varieties in increments of 20 were randomly drawn 50 times from the Haploview dataset to calculate alleles per locus.
Supplemental Figure S10. Pedigree diagram for the 12 pre61 (blue circles) and the 23 pos61 (dark purple circles) Northern winter varieties in the study panel, visualized using Helium (https://github.com/cardinalb/helium‐docs/wiki). Diameter of the circle corresponds with the relative contribution of the variety in the pedigrees of other varieties.
Supplemental Table S1. Wheat variety panel used for the study.
Supplemental Table S2. Analysis of molecular variance based on various a priori populations of U.S. wheat varieties.
Supplemental Table S3. Analysis of molecular variance based on regional wheat populations of the U.S.
Supplemental Table S4. Analysis of molecular variance based on wheat market classes.
Supplemental Table S5. Genetic diversity (PIC) summaries of U.S. wheat populations.
Supplemental Table S6. Number of haplotype alleles observed in U.S. wheat varieties from different periods.

## Data Availability

The relevant genotyping datasets and the R scripts used for analysis with documentation are available online (https://datadryad.org/stash/share/21PQi8_vI8ThdeFSXxJZ8pwYb‐6LIPR8lcAC3Z5kvSw).
