# Supplementary material for: Population structure and genetic diversity of U.S. wheat varieties
Source: Plant Genome. 2022 Mar 10;15(2):e20196. doi: 10.1002/tpg2.20196 (PMC12807204; doi:10.1002/tpg2.20196)
Supplement: Supplementary file 1 [file TPG2-15-e20196-s001.docx]

Supplemental Materials

Population Structure and Genetic Diversity of U.S. Wheat Varieties

Sajal R. Sthapit, Travis M. Ruff, Marcus A. Hooker, Deven R. See*

S.R. Sthapit, M.A. Hooker, and D.R. See, Dep. of Plant Pathology, Washington State Univ., Pullman, WA 99164; S.R. Sthapit, The Land Institute, 2440 E Water Well Rd, Salina, KS 67401; T.M. Ruff and D.R. See, USDA-ARS Wheat Health, Genetics, and Quality Research Unit, Washington State Univ., Pullman, WA 99164. *Corresponding author (deven_see@wsu.edu).

# Supplemental Figures

Supplemental Figure S1. Principal component biplots of U.S. wheat varieties coded by growth habit, kernel hardness, and kernel color.

Supplemental Figure S2. Principal component biplots of U.S. wheat varieties by market classes. HRS: hard red spring, HRW: hard red winter, HWS: hard white spring, HWW: hard white winter, SRW: soft red winter, SWS: soft white spring, SWW: soft white winter, xxW: winter varieties of unknown market class.

Supplemental Figure S3. A) Principal component biplots of U.S. wheat varieties by regions. Growth habit is denoted by the point shape and market class is denoted by color. HRS, hard red spring; HRW, hard red winter; HWS, hard white spring; HWW, hard white winter; SRW, soft red winter; SWS, soft white spring; SWW, soft white winter; xxW, winter varieties of unknown market class.

Supplemental Figure S4. Principal component biplots of U.S. wheat varieties from different time periods.

(a) **Spring**

(b) **Winter**

Supplemental Figure S5. Population structure of a) spring and b) winter varieties using 396 haplotype loci with varieties grouped by market class and region. Each vertical line represents a variety with the color representing its membership coefficient to the inferred clusters in STRUCTURE. ΔK peaked at 3 clusters for spring and 2 clusters for winter varieties. HRS, hard red spring; HWS, hard white spring; SWS, soft white spring; HRW, hard red winter; SRW, soft red winter; SWW, soft white winter; xxW, winter varieties of unknown market class.

Supplemental Figure S6. Violin plots, box plots, and mean (red dot) values of PIC distribution of U.S. wheat varieties using a) SNP and b) haplotype datasets for the genomes A, B, D, and the whole genome ABD. All SNPs used for this analysis had minor allele frequency ≥ .05 on the entire panel. Details about the datasets used are provided in the methods section.

Supplemental Figure S7. Temporal trends in the polymorphism information content (PIC) of 10,391 SNP loci in a) both spring and winter varieties, b) spring varieties, c) winter varieties, and d) market classes. The red and blue dots are the mean and median PIC values for the 30-variety sliding windows.

Supplemental Figure S8. Temporal trends in the polymorphism information content (PIC) of 1585 haplotype loci in a) both spring and winter varieties, b) spring varieties, c) winter varieties, and d) market classes. The red and blue dots are the mean and median PIC values for the 30-variety sliding windows.

Supplemental Figure S9. Means (red dot), box plots and violin plots (gray) of haplotype alleles per locus based on subpopulation size. Subpopulations of 20 to 500 varieties in increments of 20 were randomly drawn 50 times from the Haploview dataset to calculate alleles per locus.

Supplemental Figure S10. Pedigree diagram for the 12 pre-1961 (blue circles) and the 23 post-1961 (dark purple circles) Northern winter varieties in the study panel, visualized using Helium (https://github.com/cardinalb/helium-docs/wiki). Diameter of the circle corresponds with the relative contribution of the variety in the pedigrees of other varieties.

# Supplemental Tables

Supplemental Table S1. Wheat variety panel used for the study.

| **Variety** | **ACNO^a^** | **Year^b^** | **State** | **Region** | **Class** | **Headtype** | **Habit** |
| --- | --- | --- | --- | --- | --- | --- | --- |
| 711 | PI486142 | 1984 | NE | Great Plains | HRS | common | spring |
| 715 | PI486143 | 1981 | NE | Great Plains | HRS | common | spring |
| 751 | PI486144 | 1982 | NE | Great Plains | HRS | common | spring |
| 771 | PI486145 | 1980 | CA | Pacific | HRS | common | spring |
| 775 | PI601334 | 1986 | CA | Pacific | HRS | common | spring |
| 830 | PI486148 | 1982 | NE | Great Plains | HRW | common | winter |
| 835 | PI486149 | 1981 | NE | Great Plains | HRW | common | winter |
| 2154 | PI601366 | 1987 | KS | Great Plains | HRW | common | winter |
| 2157 | PI601070 | 1984 | IA | Eastern | HRW | common | winter |
| 2158 | PI601723 | 1990 | KS | Great Plains | HRW | common | winter |
| 2163 | PI601722 | 1989 | KS | Great Plains | HRW | common | winter |
| 2165 | PI601069 | 1984 | KS | Great Plains | HRW | common | winter |
| 2172 | PI601071 | 1984 | KS | Great Plains | HRW | common | winter |
| 2369 | PI600932 | 1982 | MN | Northern | HRS | common | spring |
| 2375 | PI601477 | 1989 | MN | Northern | HRS | common | spring |
| 2510 | PI550696 | 1991 | IN | Eastern | SRW | common | winter |
| 2545 | PI550697 | 1991 | IN | Eastern | SRW | common | winter |
| 2551 | PI601207 | 1985 | IN | Eastern | SRW | common | winter |
| 2552 | PI566924 | 1993 | IN | Eastern | SRW | common | winter |
| 2553 | PI601103 | 1982 | IN | Eastern | SRW | common | winter |
| 2555 | PI532914 | 1986 | IA | Eastern | SRW | common | winter |
| 2566 | PI561199 | 1993 | SC | Eastern | SRW | common | winter |
| 2571 | PI561200 | 1992 | SC | Eastern | SRW | common | winter |
| 4555 | CItr598213 | 1977 | KS | Great Plains | HRW | common | winter |
| 4578 | CItr599987 | 1978 | KS | Great Plains | HRW | common | winter |
| 5221 | CItr598214 | 1976 | KS | Great Plains | HRW | common | winter |
| 5232 | CItr598212 | 1976 | KS | Great Plains | HRW | common | winter |
| 5411 | CItr598210 | 1973 | KS | Great Plains | HRW | common | winter |
| 5422 | CItr595216 | 1977 | KS | Great Plains | HRW | common | winter |
| 5466 | CItr596624 | 1978 | KS | Great Plains | HRW | common | winter |
| 5469 | PI601249 | 1985 | KS | Great Plains | HRW | common | winter |
| 5630 | PI601248 | 1985 | KS | Great Plains | HRW | common | winter |
| 7805 | PI601606 | 1988 | KS | Great Plains | HRW | common | winter |
| 7833 | PI601607 | 1988 | KS | Great Plains | HRW | common | winter |
| 7837 | PI601608 | 1988 | KS | Great Plains | HRW | common | winter |
| 2737W | PI561197 | 1992 | IN | Eastern | SWW | common | winter |
| A99AR | PI600923 | 1982 | MN | Northern | HRS | common | spring |
| Abe | CItr15375 | 1972 | IN | Eastern | SRW | common | winter |
| Abilene | PI511307 | 1987 | KS | Great Plains | HRW | common | winter |
| Ace | CItr13384 | 1960 | AR | Eastern | SRW | common | winter |
| Adams | CItr13722 | 1968 | OR | PNW | HWS | common | spring |
| Adena | PI481852 | 1984 | OH | Eastern | SRW | common | winter |
| Admire | PI583810 | 1994 | AZ | Pacific | HRS | common | spring |
| Agassiz | PI478771 | 1983 | ND | Northern | HRW | common | winter |
| Agent | CItr13523 | 1967 | OK | Great Plains | HRW | common | winter |
| Agrus | CItr13228 | 1972 | IN | Eastern | SRW | common | winter |
| Akron | PI584504 | 1994 | CO | Great Plains | HRW | common | winter |
| Albit | CItr8275 | 1926 | WA | PNW | SWW | club | winter |
| Alex | PI17910 | 1981 | ND | Northern | HRS | common | spring |
| Alice | PI644223 | 2006 | SD | Northern | HWW | common | winter |
| Alicel | CItr11700 | 1932 | OR | PNW | SWW | club | winter |
| Allegiance | PI641952 | 2002 | KY | Eastern | SRW | common | winter |
| Alliance | PI573096 | 1993 | NE | Great Plains | HRW | common | winter |
| Alpowa | PI566596 | 1994 | WA | PNW | SWS | common | spring |
| Amidon | PI527682 | 1988 | ND | Northern | HRS | common | spring |
| Anderson | CItr12536 | 1954 | SC | Eastern | SRW | common | winter |
| Andnox | CItr13907 | 1966 | SC | Eastern | SRW | common | winter |
| Andrews | PI512282 | 1987 | WA | PNW | HRW | common | winter |
| Angus | CItr17744 | 1978 | MN | Northern | HRS | common | spring |
| Ankor | PI632275 | 2002 | CO | Great Plains | HRW | common | winter |
| Antelope | PI633910 | 2003 | NE | Great Plains | HWW | common | winter |
| Anza | CItr15284 | 1971 | CA | Pacific | HRS | common | spring |
| Apex 83 | PI601073 | 1983 | AZ | Pacific | HRS | common | spring |
| AR 910 | PI636470 | 2004 | AR | Eastern | SRW | common | winter |
| Arapahoe | PI518591 | 1988 | NE | Great Plains | HRW | common | winter |
| Arcadian | CItr565362 | 1895 | NY | Eastern | SWW | common | winter |
| Archer | CItr17940 | 1981 | CO | Great Plains | HRW | common | winter |
| Arco | CItr8246 | 1928 | OR | PNW | SWW | common | winter |
| Ark | CItr15286 | 1972 | ID | PNW | HRW | common | winter |
| Arkan | PI475771 | 1982 | KS | Great Plains | HRW | common | winter |
| Arkansas No. 105 | CItr565390 | 1918 | AR | Eastern | SRW | common | winter |
| Arrow | CItr15079 | 1971 | NY | Eastern | SWW | common | winter |
| Arrowsmith | PI633911 | 2003 | NE | Great Plains | HWW | common | winter |
| ARS-Amber | PI665047 | 2012 | WA | PNW | SWW | common | winter |
| ARS-Chrystal | PI665049 | 2012 | WA | PNW | SWW | club | winter |
| ARS-Crescent | PI665048 | 2012 | WA | PNW | SWW | club | winter |
| ARS-Selbu | PI667744 | 2013 | WA | PNW | SWW | common | winter |
| Arthur | CItr14425 | 1968 | IN | Eastern | SRW | common | winter |
| Arthur 71 | CItr15282 | 1971 | IN | Eastern | SRW | common | winter |
| Ashkof | CItr6680 | 1923 | WI | Eastern | HRW | common | winter |
| Athena | CItr11693 | 1931 | OR | PNW | SWW | common | winter |
| Atlas 50 | CItr12534 | 1948 | NC | Eastern | SRW | common | winter |
| Atlas 66 | CItr12561 | 1948 | NC | Eastern | SRW | common | winter |
| ATW 270 | PI601806 | 1989 | FL | Eastern | SRW | common | winter |
| Auburn | PI17898 | 1981 | IN | Eastern | SRW | common | winter |
| Augusta | CItr17831 | 1979 | MI | Eastern | SWW | common | winter |
| Austin | CItr12346 | 1942 | TX | Great Plains | SRW | common | winter |
| Avalanche | PI620766 | 2001 | CO | Great Plains | HWW | common | winter |
| Avoca | CItr13395 | 1954 | OK | Great Plains | HRW | common | winter |
| Awned Onas | CItr12235 | 1950 | CA | Pacific | SWS | common | spring |
| Awnless Baart | CItr12236 | 1942 | CA | Pacific | HWS | common | spring |
| Aztec | CItr13016 | 1958 | NM | Great Plains | HRW | common | winter |
| Baart 38 | CItr11907 | 1939 | CA | Pacific | HWS | common | spring |
| Baart 46 | CItr12386 | 1948 | CA | Pacific | HWS | common | spring |
| Baca | CItr15891 | 1973 | CO | Great Plains | HRW | common | winter |
| BacUp | PI596533 | 1996 | MN | Northern | HRS | common | spring |
| Bailey 4287 | PI600859 | 1982 | IN | Eastern | SRW | common | winter |
| Baker | PI573002 | 1987 | AZ | Pacific | HRS | common | spring |
| Baldrock | CItr11538 | 1931 | MI | Eastern | SRW | common | winter |
| Bannock | CItr15318 | 1972 | ID | PNW | HRS | common | spring |
| Barbee | CItr17417 | 1976 | WA | PNW | SWW | club | winter |
| Basin | PI601238 | 1984 | WA | PNW | SWW | common | winter |
| Beau | CItr17420 | 1976 | IN | Eastern | SRW | common | winter |
| Bennett | CItr17723 | 1978 | NE | Great Plains | HRW | common | winter |
| Bergen | PI538768 | 1990 | CO | Great Plains | HRS | common | spring |
| Big Club 37 | CItr11901 | 1937 | CA | Pacific | SWS | club | spring |
| Bison | CItr12518 | 1956 | KS | Great Plains | HRW | common | winter |
| Blackhawk | CItr12218 | 1944 | WI | Eastern | SRW | common | winter |
| Blackhull | CItr6251 | 1917 | KS | Great Plains | HRW | common | winter |
| Blanca | PI501533 | 1986 | ID | PNW | SWS | common | spring |
| Blizzard | PI512302 | 1989 | ID | PNW | HRW | common | winter |
| Blount's Lambrigg | CItr42207 | 1916 | CO | Great Plains | xxW | common | winter |
| Blue Jacket | CItr12502 | 1946 | KS | Great Plains | HRW | common | winter |
| Blueboy | CItr14031 | 1967 | NC | Eastern | SRW | common | winter |
| Bonanza | CItr14077 | 1969 | KS | Great Plains | HRS | common | spring |
| Brill | CItr11853 | 1936 | IL | Eastern | HRW | common | winter |
| Bronze | CItr14013 | 1974 | SD | Northern | HRW | common | winter |
| Brule | PI466739 | 1981 | NE | Great Plains | HRW | common | winter |
| Buckshot | PI481541 | 1983 | CO | Great Plains | HRS | common | spring |
| Butler | CItr12527 | 1947 | OH | Eastern | SRW | common | winter |
| Caddo | CItr13536 | 1963 | TX | Great Plains | HRW | common | winter |
| Calorwa | PI566594 | 1994 | WA | PNW | SWS | club | spring |
| Canawa | CItr11854 | 1935 | WV | Eastern | SRW | common | winter |
| Capitan | CItr17591 | 1978 | NM | Great Plains | HRW | common | winter |
| Carleeds | CItr11801 | 1936 | ND | Northern | HRS | common | spring |
| Carlson's Fife | CItr11922 | 1941 | WA | PNW | SRW | common | winter |
| Carson | PI501534 | 1986 | CO | Great Plains | HRW | common | winter |
| Cataldo | PI642361 | 2007 | ID | PNW | SWS | common | spring |
| Celtic | PI502966 | 1986 | CO | Great Plains | HRS | common | spring |
| Centennial | PI537303 | 1990 | ID | PNW | SWS | common | spring |
| Cheney | CItr17765 | 1978 | KS | Great Plains | HRW | common | winter |
| Chiefkan | CItr11754 | 1935 | KS | Great Plains | HRW | common | winter |
| Clark | PI512337 | 1987 | IN | Eastern | SRW | common | winter |
| Clemson 201 | PI585044 | 1994 | SC | Eastern | SRW | common | winter |
| Cloud | CItr17276 | 1973 | KS | Great Plains | HRW | common | winter |
| Coastal | CItr12463 | 1949 | SC | Eastern | SRW | common | winter |
| Coker 47-27 | CItr12563 | 1950 | SC | Eastern | SRW | common | winter |
| Coker 61-19 | CItr13783 | 1960 | SC | Eastern | SRW | common | winter |
| Coker 68-15 | CItr15291 | 1971 | SC | Eastern | SRW | common | winter |
| Coker 762 | PI17924 | 1980 | SC | Eastern | SRW | common | winter |
| Collin | PI511849 | 1986 | TX | Great Plains | HRW | common | winter |
| Colorado No. 50 | CItr4959 | 1909 | CO | Great Plains | SWS | common | spring |
| Colorow | CItr12865 | 1960 | CO | Great Plains | HRW | common | winter |
| Colt | PI476975 | 1983 | NE | Great Plains | HRW | common | winter |
| Comet | CItr11465 | 1940 | MT | Northern | HRS | common | spring |
| Concho | CItr12517 | 1954 | OK | Great Plains | HRW | common | winter |
| Copper | PI502644 | 1986 | ID | PNW | HRS | common | spring |
| Coteau | CItr17749 | 1978 | ND | Northern | HRS | common | spring |
| Courtney | PI476848 | 1983 | CA | Pacific | HRS | common | spring |
| Crew | PI17951 | 1982 | WA | PNW | SWW | club | winter |
| Dalen | PI552812 | 1991 | CO | Great Plains | HRS | common | spring |
| Dancer | PI17890 | 1980 | CO | Great Plains | SRW | common | winter |
| Delmar | CItr13442 | 1961 | UT | PNW | HRW | common | winter |
| Deloris | PI631447 | 2002 | UT | PNW | HRW | common | winter |
| Delta Queen | CItr17893 | 1978 | SC | Eastern | SRW | common | winter |
| Dirkwin | CItr17745 | 1978 | ID | PNW | SWS | common | spring |
| DK-49S | CItr595215 | 1978 | TX | Great Plains | HRS | common | spring |
| Dodge | PI506344 | 1986 | KS | Great Plains | HRW | common | winter |
| Downy | CItr17421 | 1976 | IN | Eastern | SRW | common | winter |
| Duke | PI17856 | 1981 | CO | Great Plains | HRW | common | winter |
| Dutro Clipper | CItr10035 | 1930 | IN | Eastern | xxW | common | winter |
| Eagle Chief | CItr8868 | 1927 | OK | Great Plains | HRW | common | winter |
| Early Blackhull | CItr8856 | 1928 | KS | Great Plains | HRW | common | winter |
| Early Defiance | CItr6480 | 1878 | CA | Pacific | SWS | common | spring |
| Early Premium | CItr11858 | 1937 | MO | Eastern | SRW | common | winter |
| Edwall | PI477919 | 1984 | WA | PNW | SWS | common | spring |
| Edwin | PI606765 | 1999 | WA | PNW | SWW | common | winter |
| Eickmeyer | CItr12035 | 1939 | WA | PNW | SWW | common | winter |
| Eider | PI478896 | 1983 | IN | Eastern | SRW | common | winter |
| Eklund | CItr614656 | 1976 | MN | Northern | HRW | common | winter |
| Elgin | CItr11755 | 1942 | OR | PNW | SWW | common | winter |
| Ellar | CItr17289 | 1974 | ND | Northern | HRS | common | spring |
| Elmar | CItr12392 | 1949 | WA | PNW | SWW | common | winter |
| Eltan | PI536994 | 1990 | WA | PNW | SWW | common | winter |
| Encore | PI600839 | 1981 | KS | Great Plains | HRW | common | winter |
| Enid | CItr11508 | 1930 | OK | Great Plains | HRW | common | winter |
| Era | CItr13986 | 1970 | MN | Northern | HRS | common | spring |
| Erhardt | PI564761 | 1996 | MT | Northern | HRW | common | winter |
| Erik | PI476849 | 1983 | CO | Great Plains | HRS | common | spring |
| Escondido | CItr8240 | 1928 | CA | Pacific | SWS | common | spring |
| Escondido 41 | CItr12239 | 1942 | CA | Pacific | SWS | common | spring |
| Eureka | CItr17738 | 1978 | SD | Northern | HRS | common | spring |
| Excel | PI555465 | 1990 | OH | Eastern | SRW | common | winter |
| Exchange | CItr12635 | 1960 | IN | Eastern | xxW | common | winter |
| Express | PI573003 | 1991 | AZ | Pacific | HRS | common | spring |
| Extra Early Blackhull | CItr13664 | 1951 | TX | Great Plains | HRW | common | winter |
| F.S. 401 | PI476850 | 1983 | IN | Eastern | SRW | common | winter |
| Fairfield | CItr12013 | 1942 | IN | Eastern | SRW | common | winter |
| Faro | CItr17590 | 1976 | OR | PNW | SWW | common | winter |
| Federation 41 | CItr12230 | 1942 | CA | Pacific | SWS | common | spring |
| Federation 41M | CItr12391 | 1942 | WA | PNW | HWS | common | spring |
| Federation 67 | CItr13732 | 1967 | ID | PNW | SWS | common | spring |
| Feland | PI600924 | 1983 | SC | Eastern | SRW | common | winter |
| FFR 555W | PI560318 | 1991 | IN | Eastern | SRW | common | winter |
| Fielder | CItr17268 | 1974 | ID | PNW | SWS | common | spring |
| Fieldwin | CItr17425 | 1977 | ID | PNW | SWS | common | spring |
| Fillmore | PI469272 | 1982 | IN | Eastern | SRW | common | winter |
| Fineway | PI653509 | 2008 | WA | PNW | HRW | common | winter |
| Finley | PI586757 | 1995 | WA | PNW | HRW | common | winter |
| Fjeld | PI531243 | 1989 | KS | Great Plains | HRS | common | spring |
| Fletcher | CItr13985 | 1970 | MN | Northern | HRS | common | spring |
| Florida 302 | PI601163 | 1984 | FL | Eastern | SRW | common | winter |
| Florida 303 | PI601807 | 1988 | FL | Eastern | SRW | common | winter |
| Florida 304 | PI562528 | 1992 | FL | Eastern | SRW | common | winter |
| Fortuna | CItr13596 | 1966 | ND | Northern | HRS | common | spring |
| Forward | CItr11392 | 1920 | NY | Eastern | SRW | common | winter |
| Forx | CItr14126 | 1964 | ND | Northern | HRS | common | spring |
| Fox | CItr13987 | 1970 | TX | Great Plains | HRW | common | winter |
| Frankenmuth | CItr17830 | 1979 | MI | Eastern | SWW | common | winter |
| Franklin | CItr15317 | 1972 | ID | PNW | HRW | common | winter |
| Freedom | PI562382 | 1991 | OH | Eastern | SRW | common | winter |
| Fremont | CItr14056 | 1970 | UT | PNW | HRS | common | spring |
| Frisco | CItr13106 | 1953 | TX | Great Plains | SRW | common | winter |
| Frontiersman | PI600832 | 1981 | KS | Great Plains | HRW | common | winter |
| Fulcaster | CItr4862 | 1886 | MD | Eastern | SRW | common | winter |
| Fulcaster 612 | CItr13715 | 1940 | TN | Eastern | SRW | common | winter |
| Fulhard | CItr8257 | 1907 | KS | Great Plains | HRW | common | winter |
| Fulhio | CItr6999 | 1920 | OH | Eastern | SRW | common | winter |
| Fulton | CItr13358 | 1964 | OH | Eastern | SRW | common | winter |
| Funk Seeds 7171 | CItr17403 | 1975 | IL | Eastern | xxW | common | winter |
| Funk Seeds 7172 | CItr17404 | 1975 | IL | Eastern | xxW | common | winter |
| Funk Seeds 7174 | CItr17405 | 1975 | IL | Eastern | xxW | common | winter |
| Funk W-332 | CItr17244 | 1972 | IL | Eastern | HRW | common | winter |
| Funk W-335 | CItr17350 | 1975 | IL | Eastern | HRW | common | winter |
| Funk W-433 | CItr17245 | 1972 | AZ | Pacific | HRS | common | spring |
| Funk W-504 | CItr17351 | 1974 | IL | Eastern | SRW | common | winter |
| Fuzz | CItr17412 | 1975 | IN | Eastern | SRW | common | winter |
| GA-Andy | PI561843 | 1990 | GA | Eastern | SRW | common | winter |
| Gage | CItr13532 | 1963 | NE | Great Plains | HRW | common | winter |
| GA-Gore | PI561842 | 1990 | GA | Eastern | SRW | common | winter |
| Gaines | CItr13448 | 1961 | WA | PNW | SWW | common | winter |
| Gasser | CItr13289 | 1955 | AK | Alaska | HRS | common | spring |
| Gasta | CItr11398 | 1931 | GA | Eastern | SRW | common | winter |
| GB 2148 | CItr595212 | 1975 | KS | Great Plains | HRW | common | winter |
| Gene | PI560129 | 1992 | OR | PNW | SWW | common | winter |
| Genro | CItr11535 | 1928 | WA | PNW | SWW | common | winter |
| Gent | CItr17293 | 1974 | SD | Northern | HRW | common | winter |
| Georgia 100 | PI538257 | 1988 | GA | Eastern | SRW | common | winter |
| Glacier | PI555586 | 1991 | WI | Eastern | SRW | common | winter |
| Glenman | PI483235 | 1985 | MT | Northern | HRS | common | spring |
| Glory | PI592750 | 1992 | OH | Eastern | SRW | common | winter |
| Glyndon | CItr25991 | 1898 | MN | Northern | HRS | common | spring |
| Golden | CItr10063 | 1930 | OR | PNW | SWW | common | winter |
| Golden 50 | CItr14190 | 1963 | KS | Great Plains | HRW | common | winter |
| Golden Cross | CItr5180 | 1888 | NY | Eastern | SRW | common | winter |
| Goldenchief | CItr14487 | 1968 | KS | Great Plains | HRW | common | winter |
| Goodstreak | PI632434 | 2002 | NE | Great Plains | HRW | common | winter |
| Goss | CItr12870 | 1949 | KS | Great Plains | HRW | common | winter |
| GR855 | PI508286 | 1985 | OH | Eastern | SRW | common | winter |
| GR876 | PI515951 | 1987 | OH | Eastern | SRW | common | winter |
| Grandin | PI531005 | 1989 | ND | Northern | HRS | common | spring |
| Grandprize | CItr4876 | 1910 | NY | Eastern | SRW | common | winter |
| Grant | PI572548 | 1993 | IN | Eastern | SRW | common | winter |
| Greer | CItr17725 | 1978 | ID | PNW | SWW | common | winter |
| Guard | PI17934 | 1983 | SD | Northern | HRS | common | spring |
| Gus | PI531004 | 1989 | ND | Northern | HRS | common | spring |
| Gypsum | CItr4762 | 1912 | CO | Great Plains | SWS | common | spring |
| Hadden | CItr13488 | 1962 | SC | Eastern | SRW | common | winter |
| Hallam | PI638790 | 2005 | NE | Great Plains | HRW | common | winter |
| Halt | PI584505 | 1994 | CO | Great Plains | HRW | common | winter |
| Hancock | PI531197 | 1988 | KS | Great Plains | SRW | common | winter |
| Hansel | CItr17296 | 1974 | UT | PNW | HRW | common | winter |
| Hard Baart | CItr8274 | 1922 | AZ | Pacific | HWS | common | spring |
| Hard Federation 31 | CItr8255 | 1928 | OR | PNW | SWS | common | spring |
| Harding | PI608049 | 1999 | CO | Great Plains | HRW | common | winter |
| Hardired | CItr12411 | 1940 | SC | Eastern | SRW | common | winter |
| Harry | PI632435 | 2002 | NE | Great Plains | HRW | common | winter |
| Hart | CItr17426 | 1976 | MO | Eastern | SRW | common | winter |
| Harvest Queen | CItr5314 | 1897 | KS | Great Plains | SRW | common | winter |
| Hatcher | PI638512 | 2004 | CO | Great Plains | HRW | common | winter |
| Hatton | CItr17772 | 1979 | WA | PNW | HRW | common | winter |
| Haven | PI547082 | 1991 | KS | Great Plains | HRW | common | winter |
| Hayden | PI608040 | 1999 | CO | Great Plains | HRW | common | winter |
| Haynes Bluestem | CItr2874 | 1895 | ND | Northern | HRS | common | spring |
| Heglar | CItr17269 | 1974 | ID | PNW | HRW | common | winter |
| Henry | CItr12265 | 1944 | WI | Eastern | HRS | common | spring |
| Hi-Line | PI549275 | 1991 | MT | Northern | HRS | common | spring |
| Hoff | PI560128 | 1992 | OR | PNW | HRW | common | winter |
| Honor | CItr6161 | 1920 | NY | Eastern | SWW | common | winter |
| Hope | CItr8178 | 1927 | SD | Northern | HRS | common | spring |
| Hosar | CItr10067 | 1930 | OR | PNW | xxW | common | winter |
| HR 53 | PI478009 | 1983 | CO | Great Plains | HRW | common | winter |
| HR 64 | PI478010 | 1983 | CO | Great Plains | HRW | common | winter |
| Hume | CItr13526 | 1965 | SD | Northern | HRW | common | winter |
| Hunter | PI468977 | 1982 | SC | Eastern | SRW | common | winter |
| Hybrid 143 | CItr4160 | 1907 | WA | PNW | SWS | common | spring |
| Hybrid 63 | CItr4510 | 1907 | WA | PNW | SWS | common | spring |
| Hymar | CItr11605 | 1935 | WA | PNW | SWW | common | winter |
| Hyper | CItr8875 | 1929 | WA | PNW | SWS | common | spring |
| Idaed | CItr11706 | 1938 | ID | PNW | SWS | common | spring |
| Idaed 59 | CItr13631 | 1962 | ID | PNW | SWS | common | spring |
| IDO644 | PI660549 | 2011 | ID | PNW | SWS | common | spring |
| IDO671 | PI660548 | 2011 | ID | PNW | SWS | common | spring |
| IDO686 | PI660551 | 2011 | ID | PNW | SWS | common | spring |
| IDO687 | PI660552 | 2011 | ID | PNW | SWS | common | spring |
| III | CItr598209 | 1974 | KS | Great Plains | HRW | common | winter |
| Illini Chief | CItr5406 | 1915 | IL | Eastern | SRW | common | winter |
| Illinois No. 2 | CItr11537 | 1931 | IL | Eastern | SRW | common | winter |
| Imbler | CItr10066 | 1930 | OR | PNW | SWW | common | winter |
| Indian | CItr4489 | 1915 | ID | PNW | SWS | common | spring |
| Ingal | CItr562647 | 1955 | AK | Alaska | HRS | common | spring |
| Inia 66R | CItr15328 | 1969 | CA | Pacific | HRS | common | spring |
| Intrada | PI631402 | 2000 | OK | Great Plains | HWW | common | winter |
| InW0731 | PI652933 | 2007 | IN | Eastern | SRW | common | winter |
| Inw8841 | PI531247 | 1989 | IN | Eastern | SRW | common | winter |
| Inw8852 | PI531248 | 1989 | IN | Eastern | SRW | common | winter |
| Inw9241 | PI583826 | 1994 | IN | Eastern | SRW | common | winter |
| Iobred | CItr6934 | 1923 | IA | Eastern | HRW | common | winter |
| Iobred 73 | CItr583669 | 1962 | IA | Eastern | HRW | common | winter |
| Iohardi | CItr12510 | 1948 | IA | Eastern | HRW | common | winter |
| Iona | PI618734 | 1999 | ID | PNW | HRS | common | spring |
| Ionia | CItr14469 | 1969 | MI | Eastern | SWW | common | winter |
| Iowin | CItr10017 | 1930 | IA | Eastern | HRW | common | winter |
| Irwin Dicklow | CItr8855 | 1912 | ID | PNW | SWS | common | spring |
| Itana | CItr12933 | 1956 | MT | Northern | HRW | common | winter |
| Jacmar | CItr608016 | 1978 | WA | PNW | SWW | common | winter |
| James | CItr17791 | 1979 | SD | Northern | HRS | common | spring |
| Jeff | CItr17270 | 1974 | ID | PNW | HRW | common | winter |
| Jefferson | PI603040 | 1998 | ID | PNW | HRS | common | spring |
| Jerry | PI632433 | 2001 | ND | Northern | HRW | common | winter |
| John | PI494095 | 1984 | WA | PNW | SWW | common | winter |
| Joleen | CItr17411 | 1975 | KS | Great Plains | HRW | common | winter |
| Jones Fife | CItr4468 | 1889 | NY | Eastern | SRW | common | winter |
| Jubilee | PI614839 | 2001 | ID | PNW | SWS | common | spring |
| Judith | PI584526 | 1989 | MT | Northern | HRW | common | winter |
| Juniper | PI639951 | 2006 | ID | PNW | HRW | common | winter |
| Justin | CItr13462 | 1962 | ND | Northern | HRS | common | spring |
| Kancom | CItr13362 | 1956 | TN | Eastern | SRW | common | winter |
| Kanhull | CItr11877 | 1935 | KS | Great Plains | HRW | common | winter |
| KanKing | CItr12719 | 1952 | KS | Great Plains | HRW | common | winter |
| Kanqueen | CItr12762 | 1949 | KS | Great Plains | SRW | common | winter |
| Kanred | CItr5146 | 1917 | KS | Great Plains | HRW | common | winter |
| Karmont | CItr6700 | 1921 | MT | Northern | HRW | common | winter |
| Kaw 61 | CItr12871 | 1960 | KS | Great Plains | HRW | common | winter |
| Kawvale | CItr8180 | 1929 | KS | Great Plains | HRW | common | winter |
| Kay | PI502970 | 1986 | IN | Eastern | SRW | common | winter |
| Kenosha | CItr14025 | 1968 | WI | Eastern | SRW | common | winter |
| Key | CItr15928 | 1976 | IN | Eastern | SRW | common | winter |
| Kiowa | CItr12133 | 1950 | KS | Great Plains | HRW | common | winter |
| Kirwin | CItr17275 | 1973 | KS | Great Plains | HRW | common | winter |
| Kitt | CItr518818 | 1975 | MN | Northern | HRS | common | spring |
| Klasic | PI486139 | 1982 | NE | Great Plains | HWS | common | spring |
| Kmor | PI536995 | 1990 | WA | PNW | SWW | common | winter |
| Knox 62 | CItr13701 | 1962 | IN | Eastern | SRW | common | winter |
| Komar | CItr8004 | 1930 | ND | Northern | HRS | common | spring |
| Krona | PI564087 | 1992 | CO | Great Plains | HRS | common | spring |
| Kruse | CItr11524 | 1957 | MT | Northern | SRW | common | winter |
| Lafron | CItr14022 | 1964 | IN | Eastern | xxW | common | winter |
| Lamar | PI559719 | 1988 | CO | Great Plains | HRW | common | winter |
| Lambert | PI583372 | 1994 | ID | PNW | SWW | common | winter |
| Lancer | CItr13547 | 1963 | NE | Great Plains | HRW | common | winter |
| Lani | CItr14021 | 1964 | IN | Eastern | xxW | common | winter |
| LaPorte | CItr12557 | 1957 | IN | Eastern | SRW | common | winter |
| Lark | CItr17338 | 1971 | CA | Pacific | HRS | common | spring |
| Leap | CItr4823 | 1907 | VA | Eastern | SRW | common | winter |
| Lee | CItr12488 | 1950 | MN | Northern | HRS | common | spring |
| Leif | PI601206 | 1985 | MN | Northern | HRS | common | spring |
| Lemhi | CItr11415 | 1939 | ID | PNW | SWS | common | spring |
| Lemhi 53 | CItr13258 | 1953 | ID | PNW | SWS | common | spring |
| Lemhi 62 | CItr13435 | 1962 | ID | PNW | SWS | common | spring |
| Lemhi 66 | CItr13969 | 1966 | ID | PNW | SWS | common | spring |
| Len | CItr17790 | 1979 | ND | Northern | HRS | common | spring |
| Lenore | CItr17726 | 1978 | ID | PNW | SWW | common | winter |
| Lew | CItr17429 | 1976 | MT | Northern | HRS | common | spring |
| Lewis | CItr13705 | 1964 | MO | Eastern | SRW | common | winter |
| Lewjain | PI17909 | 1982 | WA | PNW | SWW | common | winter |
| LHS | PI660547 | 2011 | ID | PNW | HWW | common | winter |
| Lincoln | PI506404 | 1987 | KS | Great Plains | SRW | common | winter |
| Lindon | CItr17440 | 1975 | CO | Great Plains | HRW | common | winter |
| LoLo | PI614840 | 2000 | ID | PNW | HWS | common | spring |
| Longberry No. 1 | CItr5823 | 1898 | NY | Eastern | SWW | common | winter |
| Longhorn | PI552813 | 1991 | KS | Great Plains | HRW | common | winter |
| Louise | PI634865 | 2005 | WA | PNW | SWS | common | spring |
| Lucas | CItr12990 | 1959 | OH | Eastern | SRW | common | winter |
| Luft | CItr13729 | 1962 | ID | PNW | SWW | common | winter |
| Luke | CItr14586 | 1970 | WA | PNW | SWW | common | winter |
| Lynn | PI565359 | 1991 | CA | Pacific | HWS | common | spring |
| Mace | PI651043 | 2007 | NE | Great Plains | HRW | common | winter |
| MacVicar | PI552427 | 1992 | OR | PNW | SWW | common | winter |
| Madison | PI547041 | 1990 | VA | Eastern | SRW | common | winter |
| Madsen | PI511673 | 1988 | WA | PNW | SWW | common | winter |
| Magnum | PI477285 | 1983 | CO | Great Plains | SRW | common | winter |
| Manning | CItr17846 | 1979 | UT | PNW | HRW | common | winter |
| Marberg | PI518816 | 1980 | MT | Northern | HRS | common | spring |
| Marett Chancellor | CItr12566 | 1947 | SC | Eastern | SRW | common | winter |
| Marfed | CItr11919 | 1946 | WA | PNW | SWS | common | spring |
| Maricopa | CItr14129 | 1966 | AZ | Pacific | HWS | common | spring |
| Marshall | PI17920 | 1982 | MN | Northern | HRS | common | spring |
| Martin | CItr4463 | 1880 | NY | Eastern | SWW | common | winter |
| Martin Amber | PI565360 | 1991 | NY | Eastern | SWW | common | winter |
| Marvel | CItr8876 | 1928 | SD | Northern | SRW | common | winter |
| Massey | PI17953 | 1981 | VA | Eastern | SRW | common | winter |
| Maverick | CItr17728 | 1977 | TX | Great Plains | HRW | common | winter |
| Mayview | CItr5874 | 1915 | WA | PNW | SWS | common | spring |
| McCall | CItr13842 | 1965 | WA | PNW | HRW | common | winter |
| McGuire | PI593890 | 1996 | MT | Northern | HRW | common | winter |
| McKay | PI17903 | 1981 | ID | PNW | HRS | common | spring |
| McNair 1587 | CItr17279 | 1973 | NC | Eastern | SRW | common | winter |
| McNair 1813 | CItr15289 | 1975 | NC | Eastern | SRW | common | winter |
| McNair 2203 | CItr15228 | 1970 | NC | Eastern | SRW | common | winter |
| McNeal | PI574642 | 1994 | MT | Northern | HRS | common | spring |
| Meggie | PI486337 | 1984 | CO | Great Plains | HRW | common | winter |
| Mercury | CItr11872 | 1933 | ND | Northern | HRS | common | spring |
| Meridian | PI557013 | 1992 | ID | PNW | HRW | common | winter |
| Merit | CItr11870 | 1937 | ND | Northern | HRS | common | spring |
| Merrimac | PI535454 | 1988 | WI | Eastern | SRW | common | winter |
| Mesa | PI511308 | 1987 | KS | Great Plains | HRW | common | winter |
| Michigan Amber | CItr11379 | 1875 | IN | Eastern | SRW | common | winter |
| Michikof | CItr6990 | 1920 | IN | Eastern | HRW | common | winter |
| Mida | CItr12008 | 1944 | ND | Northern | HRS | common | spring |
| Milam | CItr13369 | 1959 | TX | Great Plains | HRS | common | spring |
| Milburn | PI601252 | 1985 | TX | Great Plains | HRW | common | winter |
| Mindoro | PI601075 | 1984 | CA | Pacific | HRS | common | spring |
| MinnPro | PI532149 | 1989 | MN | Northern | HRS | common | spring |
| Minter | CItr12138 | 1948 | MN | Northern | HRW | common | winter |
| Minturki | CItr6155 | 1919 | MN | Northern | HRW | common | winter |
| Missouri Valley | CItr10046 | 1930 | ND | Northern | HRS | common | spring |
| Moking | CItr12556 | 1946 | KS | Great Plains | SRW | common | winter |
| Monon | CItr13278 | 1959 | IN | Eastern | SRW | common | winter |
| Moran | CItr13743 | 1967 | ID | PNW | HRS | common | spring |
| Mosida | CItr6688 | 1924 | ID | PNW | HRW | common | winter |
| Nabob | CItr8869 | 1928 | OH | Eastern | SRW | common | winter |
| NE01643 | PI647959 | 2007 | NE | Great Plains | HRW | common | winter |
| NE05548 | PI670462 | 2014 | NE | Great Plains | HRW | common | winter |
| NE06545 | PI667038 | 2013 | NE | Great Plains | HRW | common | winter |
| Nebraska No. 28 | CItr5147 | 1916 | NE | Great Plains | SRW | common | winter |
| Nebraska No. 6 | CItr6249 | 1919 | NE | Great Plains | HRW | common | winter |
| Nebraska No. 60 | CItr6250 | 1918 | NE | Great Plains | HRW | common | winter |
| Nebred | CItr10094 | 1938 | NE | Great Plains | HRW | common | winter |
| Neeley | PI17860 | 1980 | ID | PNW | HRW | common | winter |
| Nekota | PI584997 | 1994 | NE | Great Plains | HRW | common | winter |
| Nelson | PI17770 | 1982 | AR | Eastern | SRW | common | winter |
| New Victory | CItr12677 | 1949 | WA | PNW | SWW | common | winter |
| Newana | CItr17430 | 1976 | MT | Northern | HRS | common | spring |
| Newcaster | CItr12528 | 1946 | IL | Eastern | SRW | common | winter |
| Newest Improved Triumph | CItr13668 | 1954 | OK | Great Plains | HRW | common | winter |
| Newthatch | CItr12318 | 1944 | MN | Northern | HRS | common | spring |
| Newton | CItr17715 | 1978 | KS | Great Plains | HRW | common | winter |
| Nicoma | CItr13874 | 1971 | OK | Great Plains | HRW | common | winter |
| Niobrara | PI584996 | 1994 | NE | Great Plains | HRW | common | winter |
| Nogal | PI562646 | 1992 | AK | Alaska | HRS | common | spring |
| Norak | PI601205 | 1984 | MN | Northern | HRS | common | spring |
| Norana | CItr15927 | 1973 | MT | Northern | HRS | common | spring |
| Nordic | PI506405 | 1986 | KS | Great Plains | HRS | common | spring |
| Nordman | CItr14127 | 1964 | ND | Northern | HRS | common | spring |
| Norseman | PI494102 | 1986 | CO | Great Plains | HRS | common | spring |
| Norwin | PI491533 | 1984 | MT | Northern | HRW | common | winter |
| Nowesta | CItr17390 | 1973 | ND | Northern | HRS | common | spring |
| Nudel | CItr12672 | 1947 | DE | Eastern | SRW | common | winter |
| Nugaines | CItr13968 | 1965 | WA | PNW | SWW | common | winter |
| Nuplains | PI605741 | 1998 | NE | Great Plains | HWW | common | winter |
| Nured | CItr12455 | 1939 | NY | Eastern | SRW | common | winter |
| NY Batavia | PI595085 | 1996 | NY | Eastern | SWW | common | winter |
| Oasis | CItr15929 | 1973 | IN | Eastern | SRW | common | winter |
| Oatka Chief | CItr591889 | 1897 | NY | Eastern | SWW | common | winter |
| OK101 | PI631493 | 2001 | OK | Great Plains | HRW | common | winter |
| Olaf | CItr15930 | 1973 | ND | Northern | HRS | common | spring |
| Omaha | CItr13015 | 1960 | NE | Great Plains | HRW | common | winter |
| Omar | CItr13072 | 1955 | WA | PNW | SWW | common | winter |
| Omega 78 | CItr17721 | 1979 | GA | Eastern | SRW | common | winter |
| Onas 41 | CItr12229 | 1942 | CA | Pacific | SWS | common | spring |
| Onas 53 | CItr13069 | 1953 | CA | Pacific | SWS | common | spring |
| Oregon Zimmerman | CItr7359 | 1921 | OR | PNW | SWS | common | spring |
| Orfed | CItr11913 | 1943 | WA | PNW | SWS | common | spring |
| Orion | PI497986 | 1985 | CO | Great Plains | SRW | common | winter |
| Oro | CItr8220 | 1927 | OR | PNW | HRW | common | winter |
| Osage | CItr17292 | 1974 | OK | Great Plains | HRW | common | winter |
| Oslo | PI17901 | 1980 | CO | Great Plains | HRS | common | spring |
| Ottawa | CItr12804 | 1960 | KS | Great Plains | HRW | common | winter |
| Oveson | PI512338 | 1986 | OR | PNW | SWW | common | winter |
| Owens | PI17904 | 1981 | ID | PNW | SWS | common | spring |
| Pacer | PI614655 | 1988 | MO | Eastern | SRW | common | winter |
| Pacific Bluestem 37 | CItr11903 | 1937 | CA | Pacific | SWS | common | spring |
| Paha | CItr14485 | 1970 | WA | PNW | SWW | common | winter |
| Palala | CItr17847 | 1979 | ND | Northern | xxW | common | winter |
| Palo Duro | CItr14584 | 1969 | KS | Great Plains | HRW | common | winter |
| Parker | CItr13285 | 1966 | KS | Great Plains | HRW | common | winter |
| Payne | CItr17717 | 1977 | OK | Great Plains | HRW | common | winter |
| Peak | CItr14587 | 1971 | ID | PNW | HRS | common | spring |
| Peak 72 | CItr15319 | 1972 | ID | PNW | HRS | common | spring |
| Peck | CItr17298 | 1974 | ID | PNW | SWW | common | winter |
| Pedigreed Blackhull No. 60 | CItr8857 | 1917 | KS | Great Plains | HRW | common | winter |
| Penawawa | PI495916 | 1985 | WA | PNW | SWS | common | spring |
| Pennoll | CItr12755 | 1951 | PA | Eastern | SRW | common | winter |
| Penquite | CItr5948 | 1858 | OH | Eastern | SRW | common | winter |
| Phoenix | CItr17962 | 1981 | CA | Pacific | HWW | common | winter |
| Pilcraw | CItr5540 | 1917 | CA | Pacific | SWS | common | spring |
| Pilot | CItr11428 | 1939 | ND | Northern | HRS | common | spring |
| PL145 | PI600840 | 1982 | KS | Great Plains | HRW | common | winter |
| Plainsman | CItr14128 | 1964 | ND | Northern | HRS | common | spring |
| Polk | CItr13773 | 1968 | MN | Northern | HRS | common | spring |
| Pomerelle | PI592983 | 1996 | ID | PNW | SWS | common | spring |
| Ponca | CItr12128 | 1951 | KS | Great Plains | HRW | common | winter |
| Pondera | PI17828 | 1980 | MT | Northern | HRS | common | spring |
| Pontiac | PI573038 | 1993 | IN | Eastern | SRW | common | winter |
| Pony | PI601203 | 1985 | NE | Great Plains | HRW | common | winter |
| Portage | CItr5654 | 1923 | OH | Eastern | SRW | common | winter |
| Portola | CItr17415 | 1975 | CA | Pacific | HRS | common | spring |
| Poso | CItr8891 | 1930 | CA | Pacific | SWS | common | spring |
| Poso 41 | CItr12240 | 1941 | CA | Pacific | SWS | common | spring |
| Poso 44 | CItr12389 | 1945 | CA | Pacific | SWS | common | spring |
| Poso 48 | CItr12691 | 1948 | CA | Pacific | SWS | common | spring |
| Potomac | CItr15292 | 1975 | VA | Eastern | SRW | common | winter |
| Powerclub | CItr8276 | 1926 | ID | PNW | SWW | common | winter |
| Prairie | CItr12069 | 1943 | IL | Eastern | SRW | common | winter |
| Prairie Red | PI605390 | 1998 | CO | Great Plains | HRW | common | winter |
| Premier | CItr11940 | 1938 | ND | Northern | HRS | common | spring |
| Pride of Genesee | CItr3365 | 1893 | NY | Eastern | SRW | common | winter |
| Probred | CItr17410 | 1974 | MN | Northern | HRS | common | spring |
| Prodax | CItr17407 | 1974 | MN | Northern | HRS | common | spring |
| Profit 75 | CItr17348 | 1975 | CA | Pacific | HRS | common | spring |
| Progress | CItr6902 | 1921 | WI | Eastern | SRW | common | winter |
| Promontory | PI555458 | 1991 | UT | PNW | HRW | common | winter |
| Prospect | PI491568 | 1988 | SD | Northern | HRS | common | spring |
| Prosperity | CItr5380 | 1890 | NY | Eastern | SRW | common | winter |
| Prospur | CItr17408 | 1975 | MN | Northern | HRS | common | spring |
| Protor | CItr17409 | 1975 | MN | Northern | HRS | common | spring |
| Prowers | PI605389 | 1997 | CO | Great Plains | HRW | common | winter |
| Prowers 99 | PI612420 | 1999 | CO | Great Plains | HRW | common | winter |
| Purcam | CItr12294 | 1951 | IN | Eastern | SRW | common | winter |
| Purcell | PI17787 | 1981 | NY | Eastern | SWW | common | winter |
| Purplestraw | PI500000 | 1985 | MD | Eastern | SRW | common | winter |
| Quality | CItr6607 | 1918 | CA | Pacific | HWS | common | spring |
| R36 | CItr583674 | 1974 | IL | Eastern | HRW | common | winter |
| Racine | CItr13172 | 1956 | WI | Eastern | SRW | common | winter |
| Raeder | CItr17418 | 1976 | WA | PNW | SWW | common | winter |
| Ram | PI477287 | 1983 | CO | Great Plains | HRW | common | winter |
| Ramona | CItr8241 | 1935 | CA | Pacific | SWS | common | spring |
| Ramona 50 | CItr12390 | 1951 | CA | Pacific | HWS | common | spring |
| Rampart | PI593889 | 1996 | MT | Northern | HRW | common | winter |
| Ranger | CItr15316 | 1972 | ID | PNW | HRW | common | winter |
| Rawhide | PI543893 | 1990 | NE | Great Plains | HRW | common | winter |
| Read | CItr6401 | 1898 | VT | Eastern | SWW | common | winter |
| Red Chief | CItr12109 | 1940 | KS | Great Plains | HRW | common | winter |
| Red Clawson | CItr565369 | 1889 | NY | Eastern | SRW | common | winter |
| Red Jacket | CItr12713 | 1950 | KS | Great Plains | HRW | common | winter |
| Red River 68 | CItr14193 | 1968 | CA | Pacific | HRS | common | spring |
| Red Rock | CItr5597 | 1914 | MI | Eastern | SRW | common | winter |
| Red Wave | CItr3500 | 1905 | NY | Eastern | SRW | common | winter |
| Redhart | CItr8898 | 1921 | SC | Eastern | SRW | common | winter |
| Redhart 4 | CItr12003 | 1949 | SC | Eastern | SRW | common | winter |
| Redhart 5 | CItr12004 | 1949 | SC | Eastern | SRW | common | winter |
| Redhull | CItr11534 | 1921 | KS | Great Plains | HRW | common | winter |
| Redland | PI502907 | 1986 | NE | Great Plains | HRW | common | winter |
| Redwin | CItr17844 | 1979 | MT | Northern | HRW | common | winter |
| Regal | CItr7364 | 1926 | OR | PNW | HRS | common | spring |
| Regenerated Defiance | CItr3703 | 1907 | CO | Great Plains | HWS | common | spring |
| Rego | CItr13181 | 1957 | MT | Northern | HRW | common | winter |
| Reliance | CItr7370 | 1926 | OR | PNW | HRS | common | spring |
| Reliant | CItr12144 | 1940 | OK | Great Plains | HRW | common | winter |
| Rely | PI542401 | 1991 | WA | PNW | SWW | common | winter |
| Rew | CItr17294 | 1974 | OR | PNW | SWW | common | winter |
| Rex | CItr10065 | 1933 | OR | PNW | SWW | common | winter |
| RHS 8232 | PI601138 | 1984 | NC | Eastern | SRW | common | winter |
| Richland | PI632399 | 2001 | NY | Eastern | SWW | common | winter |
| Rick | PI614834 | 1985 | UT | PNW | HRS | common | spring |
| Ridit | CItr6703 | 1923 | WA | PNW | HRW | common | winter |
| Riley | CItr13702 | 1965 | IN | Eastern | SRW | common | winter |
| Riley 67 | CItr14110 | 1967 | IN | Eastern | SRW | common | winter |
| Rio | CItr10061 | 1930 | OR | PNW | HRW | common | winter |
| Rio Blanco | PI531244 | 1989 | KS | Great Plains | HWW | common | winter |
| Rival | CItr11708 | 1939 | ND | Northern | HRS | common | spring |
| Rocky | CItr17879 | 1978 | CO | Great Plains | HRW | common | winter |
| Rod | PI558510 | 1992 | WA | PNW | SWW | common | winter |
| Rodco | CItr13560 | 1957 | KS | Great Plains | HRW | common | winter |
| Roedel | CItr15175 | 1963 | OR | PNW | SWW | common | winter |
| Rohde | PI562529 | 1993 | OR | PNW | SWW | common | winter |
| Roland | CItr17716 | 1977 | IL | Eastern | SRW | common | winter |
| Rose | PI17795 | 1981 | SD | Northern | HRW | common | winter |
| Rosen | PI17607 | 1980 | AR | Eastern | SRW | common | winter |
| Rosetta | CItr13363 | 1959 | OK | Great Plains | HRW | common | winter |
| Roughrider | CItr17439 | 1975 | ND | Northern | HRW | common | winter |
| RSI 220 | PI584322 | 1994 | CA | Pacific | SRW | common | winter |
| Ruddy | CItr6465 | 1919 | WA | PNW | SRW | common | winter |
| Rulo | PI578137 | 1994 | WA | PNW | SWW | common | winter |
| Rural New Yorker No. 57 | CItr3516 | 1894 | NY | Eastern | SRW | common | winter |
| Rural New Yorker No. 6 | CItr5921 | 1894 | NY | Eastern | SRW | common | winter |
| Rushmore | CItr12273 | 1949 | SD | Northern | HRS | common | spring |
| Russell | CItr12484 | 1956 | WI | Eastern | HRS | common | spring |
| S 76 | CItr17608 | 1976 | KS | Great Plains | SRW | common | winter |
| S78 | CItr600823 | 1978 | IN | Eastern | SRW | common | winter |
| Saline | CItr12674 | 1950 | IL | Eastern | SRW | common | winter |
| Salmon | PI542976 | 1990 | ID | PNW | SWW | common | winter |
| Saluda | PI480474 | 1983 | VA | Eastern | SRW | common | winter |
| Sanford | CItr12026 | 1940 | GA | Eastern | SRW | common | winter |
| Satanta | CItr14582 | 1969 | KS | Great Plains | HRW | common | winter |
| Sawmont | CItr13544 | 1965 | MT | Northern | HRW | common | winter |
| Sawtana | CItr13304 | 1961 | MT | Northern | HRS | common | spring |
| Sawtell | CItr17424 | 1977 | ID | PNW | HRS | common | spring |
| Sawyer | PI559930 | 1991 | CO | Great Plains | SRW | common | winter |
| Scout | CItr13546 | 1964 | NE | Great Plains | HRW | common | winter |
| Scoutland | CItr14075 | 1970 | NE | Great Plains | HRW | common | winter |
| Seabreeze | CItr12611 | 1945 | TX | Great Plains | SRW | common | winter |
| Sentinel | CItr17265 | 1973 | NE | Great Plains | HRW | common | winter |
| Severn | PI17939 | 1981 | MD | Eastern | SRW | common | winter |
| Sharp | PI540401 | 1990 | SD | Northern | HRS | common | spring |
| Shasta | CItr17651 | 1976 | CA | Pacific | HRS | common | spring |
| Shepherd | CItr6163 | 1923 | NY | Eastern | SRW | common | winter |
| Sheridan | CItr13586 | 1966 | MT | Northern | HRS | common | spring |
| Sherman | CItr4430 | 1928 | OR | PNW | HRW | common | winter |
| Shield | PI491570 | 1986 | SD | Northern | HRS | common | spring |
| Shortana | CItr15233 | 1971 | MT | Northern | HRS | common | spring |
| Sibley 81 | CItr10084 | 1930 | OK | Great Plains | HRW | common | winter |
| SilverSheaf | CItr2496 | 1905 | NY | Eastern | SRW | common | winter |
| Sioux | CItr12142 | 1940 | NE | Great Plains | HRW | common | winter |
| Siouxland | PI483469 | 1984 | NE | Great Plains | HRW | common | winter |
| Solar | CItr486140 | 1978 | NE | Great Plains | HRS | common | spring |
| Southern Belle | PI17894 | 1980 | SC | Eastern | SRW | common | winter |
| Spillman | PI506350 | 1987 | WA | PNW | HRS | common | spring |
| Spinkcota | CItr12375 | 1944 | SD | Northern | HRS | common | spring |
| Spokane Chief | CItr13252 | 1953 | WA | PNW | SRW | common | winter |
| SpringField | CItr14589 | 1970 | ID | PNW | SWS | common | spring |
| SR-49 | PI487294 | 1984 | CO | Great Plains | SRW | common | winter |
| Stacy | PI17861 | 1980 | GA | Eastern | SRW | common | winter |
| Stadler | CItr13704 | 1964 | MO | Eastern | SRW | common | winter |
| Stafford | CItr12706 | 1949 | KS | Great Plains | HRW | common | winter |
| Steele | PI516196 | 1988 | IN | Eastern | SRW | common | winter |
| Sterling | PI17859 | 1980 | ID | PNW | SWS | common | spring |
| Stoa | PI520297 | 1984 | ND | Northern | HRS | common | spring |
| Stoddard | CItr15925 | 1973 | MO | Eastern | SRW | common | winter |
| Sturgeon | CItr11703 | 1934 | WI | Eastern | HRS | common | spring |
| Sullivan | CItr17684 | 1977 | IN | Eastern | SRW | common | winter |
| Super Triumph | CItr13669 | 1957 | OK | Great Plains | HRW | common | winter |
| Surprise | CItr25971 | 1909 | CA | Pacific | SWS | common | spring |
| Survivor | PI509503 | 1991 | ID | PNW | HRW | common | winter |
| Susquehanna | PI474581 | 1988 | MD | Eastern | SRW | common | winter |
| Sylvan | PI578867 | 1994 | CO | Great Plains | HRS | common | spring |
| Symphony | PI583754 | 1994 | WA | PNW | SRW | common | winter |
| Syringa | PI601452 | 1988 | ID | PNW | SWW | common | winter |
| TAM 106 | CItr17827 | 1979 | TX | Great Plains | HRW | common | winter |
| TAM 109 | PI554606 | 1991 | TX | Great Plains | HRW | common | winter |
| TAM 202 | PI561933 | 1992 | TX | Great Plains | HRW | common | winter |
| TAM 300 | PI576151 | 1993 | TX | Great Plains | HRW | common | winter |
| TAM W-101 | CItr15324 | 1971 | TX | Great Plains | HRW | common | winter |
| TAM W-104 | CItr17452 | 1975 | TX | Great Plains | HRW | common | winter |
| TAM-200 | PI578255 | 1986 | TX | Great Plains | HRW | common | winter |
| Tammy | PI601288 | 1986 | CA | Pacific | HRS | common | spring |
| Tayland | CItr12761 | 1954 | MD | Eastern | SRW | common | winter |
| Taylor | CItr12461 | 1953 | VA | Eastern | SRW | common | winter |
| Taylor 49 | CItr13249 | 1956 | NC | Eastern | SRW | common | winter |
| Tecumseh | CItr17287 | 1973 | MI | Eastern | SWW | common | winter |
| Telemark | PI506406 | 1987 | KS | Great Plains | HRS | common | spring |
| Tendoy | CItr13426 | 1960 | ID | PNW | HRW | common | winter |
| Tenmarq | CItr6936 | 1932 | KS | Great Plains | HRW | common | winter |
| Terral 877 | PI564083 | 1992 | LA | Eastern | SRW | common | winter |
| Teton | CItr15244 | 1971 | MT | Northern | HRW | common | winter |
| Texred | CItr17729 | 1977 | TX | Great Plains | HRW | common | winter |
| Thatcher | CItr10003 | 1934 | MN | Northern | HRS | common | spring |
| Thunderbird | PI497988 | 1985 | CO | Great Plains | HRW | common | winter |
| Tiber | PI517194 | 1988 | MT | Northern | HRW | common | winter |
| Ticonderoga | CItr17290 | 1973 | NY | Eastern | SWW | common | winter |
| Timstein | CItr12347 | 1939 | MN | Northern | xxW | common | winter |
| Timwin | CItr13787 | 1967 | WI | Eastern | SRW | common | winter |
| Tioga | CItr17286 | 1974 | ND | Northern | HRS | common | spring |
| Todd | CItr13110 | 1956 | KY | Eastern | SRW | common | winter |
| Tracey | CItr17925 | 1979 | CA | Pacific | HRS | common | spring |
| Trailblazer | PI506407 | 1987 | KS | Great Plains | HRW | common | winter |
| Trapper | CItr13999 | 1967 | NE | Great Plains | HRW | common | winter |
| Treasure | PI468962 | 1986 | ID | PNW | SWS | common | spring |
| Triplet | CItr5408 | 1918 | WA | PNW | SRW | common | winter |
| Trison | CItr17278 | 1973 | KS | Great Plains | HRW | common | winter |
| Triumph | CItr12132 | 1940 | OK | Great Plains | HRW | common | winter |
| Trumbull | CItr5657 | 1916 | OH | Eastern | SRW | common | winter |
| Twain | PI506408 | 1987 | KS | Great Plains | SRW | common | winter |
| Twin | CItr14588 | 1971 | ID | PNW | SWS | common | spring |
| Tyee | PI17773 | 1979 | WA | PNW | SWW | common | winter |
| Tyler | PI17899 | 1980 | VA | Eastern | SRW | common | winter |
| UI Lochsa | PI639952 | 2005 | ID | PNW | HWS | common | spring |
| UI Pettit | PI642363 | 2006 | ID | PNW | SWS | common | spring |
| UI Winchester | PI642362 | 2009 | ID | PNW | HRS | common | spring |
| Union | CItr11704 | 1921 | OR | PNW | SWS | common | spring |
| Urquie | CItr17413 | 1975 | WA | PNW | SWS | common | spring |
| USU-Apogee | PI592742 | 1996 | UT | PNW | HRS | common | spring |
| Utac | CItr10045 | 1928 | UT | PNW | HWS | common | spring |
| Utah-100 | PI594920 | 1996 | UT | PNW | HRW | common | winter |
| Ute | PI490017 | 1983 | UT | PNW | HRW | common | winter |
| V.P.I. 112 | CItr11397 | 1915 | VA | Eastern | SRW | common | winter |
| V.P.I. 131 | CItr10047 | 1915 | VA | Eastern | SRW | common | winter |
| Vahart | CItr12537 | 1945 | VA | Eastern | SRW | common | winter |
| Valprize | CItr11539 | 1931 | NY | Eastern | SRW | common | winter |
| Vance | PI532150 | 1989 | MN | Northern | HRS | common | spring |
| Vandal | PI546056 | 1991 | ID | PNW | HRS | common | spring |
| Vanguard | PI593891 | 1995 | MT | Northern | HRW | common | winter |
| Vermillion | CItr12748 | 1955 | IN | Eastern | SRW | common | winter |
| Vesta | CItr11712 | 1942 | ND | Northern | HRS | common | spring |
| Victory | PI497989 | 1985 | CO | Great Plains | HRW | common | winter |
| Vigal | PI545492 | 1990 | AK | Alaska | HRS | common | spring |
| Vigo | CItr12220 | 1946 | IN | Eastern | SRW | common | winter |
| Virginia | CItr565372 | 1910 | MD | Eastern | SRW | common | winter |
| Vista | PI562653 | 1992 | NE | Great Plains | HRW | common | winter |
| Voyager | PI546478 | 1991 | KS | Great Plains | HRW | common | winter |
| W2501 | PI516200 | 1988 | CO | Great Plains | HRS | common | spring |
| W2502 | PI516201 | 1988 | CO | Great Plains | HRS | common | spring |
| Waban | CItr12992 | 1978 | IN | Eastern | SRW | common | winter |
| Waco | PI531246 | 1989 | KS | Great Plains | HRW | common | winter |
| Wadual | PI506354 | 1987 | WA | PNW | SWS | common | spring |
| Wadual 94 | PI566595 | 1993 | WA | PNW | SWS | common | spring |
| Wahoo | PI619098 | 2000 | NE | Great Plains | HRW | common | winter |
| Wakanz | PI506352 | 1987 | WA | PNW | SWS | common | spring |
| Wakeland | CItr13382 | 1959 | NC | Eastern | SRW | common | winter |
| Waldron | CItr13958 | 1969 | ND | Northern | HRS | common | spring |
| Walera | PI486141 | 1982 | NE | Great Plains | HRS | common | spring |
| Wampum | PI17691 | 1980 | WA | PNW | HRS | common | spring |
| Wanken | CItr13659 | 1961 | OK | Great Plains | xxW | common | winter |
| Wanser | CItr13844 | 1965 | WA | PNW | HRW | common | winter |
| Wared | CItr15926 | 1974 | WA | PNW | HRS | common | spring |
| Warrior | CItr13190 | 1960 | NE | Great Plains | HRW | common | winter |
| Wasatch | CItr11925 | 1944 | UT | PNW | HRW | common | winter |
| Wawawai | PI574538 | 1994 | WA | PNW | SWS | common | spring |
| Wesel | CItr13090 | 1944 | OK | Great Plains | HRW | common | winter |
| Westar | CItr12110 | 1944 | TX | Great Plains | HRW | common | winter |
| Westbred 906R | PI483455 | 1981 | AZ | Pacific | HRS | common | spring |
| Westbred 911 | PI483456 | 1981 | AZ | Pacific | HRS | common | spring |
| Westbred Aim | PI599989 | 1979 | AZ | Pacific | HRS | common | spring |
| Westbred Challenger | PI601072 | 1983 | ND | Northern | HRS | common | spring |
| Westmont | CItr12930 | 1956 | MT | Northern | HRW | common | winter |
| Weston | CItr17727 | 1978 | ID | PNW | HRW | common | winter |
| Wheaton | PI469271 | 1983 | MN | Northern | HRS | common | spring |
| Wheeler | PI17900 | 1980 | VA | Eastern | SRW | common | winter |
| White Federation 38 | CItr11906 | 1939 | CA | Pacific | SWS | common | spring |
| White Federation 59 | CItr17579 | 1976 | CA | Pacific | SWS | common | spring |
| White Federation 60 | CItr17580 | 1976 | CA | Pacific | SWS | common | spring |
| White Mediterranean | CItr11489 | 1930 | TX | Great Plains | SWW | common | winter |
| White Odessa | CItr4655 | 1928 | ID | PNW | SWW | common | winter |
| Whitebird | PI592982 | 1996 | ID | PNW | SWS | common | spring |
| Wichita | CItr11952 | 1944 | KS | Great Plains | HRW | common | winter |
| Wilbur | CItr6797 | 1919 | OR | PNW | SWS | common | spring |
| Willett | CItr13099 | 1935 | MN | Northern | xxW | common | winter |
| Williams | PI508082 | 1984 | SC | Eastern | SRW | common | winter |
| Willow Creek | PI655073 | 2005 | MT | Northern | HRW | common | winter |
| Windstar | PI597379 | 1996 | NE | Great Plains | HRW | common | winter |
| Wings | CItr17880 | 1977 | CO | Great Plains | HRW | common | winter |
| Winoka | CItr14000 | 1969 | SD | Northern | HRW | common | winter |
| Winridge | PI17902 | 1981 | MT | Northern | HRW | common | winter |
| Wisconsin Pedigree No. 2 | CItr6683 | 1918 | WI | Eastern | HRW | common | winter |
| Wissler | CItr11610 | 1933 | ND | Northern | xxW | common | winter |
| World Seeds 1 | CItr17347 | 1974 | CA | Pacific | HWS | common | spring |
| World Seeds 13 | PI17824 | 1979 | CA | Pacific | SWS | common | spring |
| World Seeds 1651 | CItr15334 | 1969 | CA | Pacific | HRS | common | spring |
| World Seeds 1809 | CItr15012 | 1971 | CA | Pacific | HRS | common | spring |
| World Seeds 1812 | CItr14585 | 1969 | CA | Pacific | HRS | common | spring |
| World Seeds 1877 | CItr14591 | 1969 | CA | Pacific | HRS | common | spring |
| World Seeds 25 | CItr17825 | 1976 | CA | Pacific | HRS | common | spring |
| World Seeds 6 | CItr17345 | 1973 | CA | Pacific | HRS | common | spring |
| Wrangler | PI477288 | 1983 | CO | Great Plains | HRW | common | winter |
| Yamhill | CItr14563 | 1969 | OR | PNW | SWW | common | winter |
| Yecora Rojo | CItr17414 | 1975 | CA | Pacific | HRS | common | spring |
| Yogo | CItr8033 | 1932 | MT | Northern | HRW | common | winter |
| Yolo | PI17961 | 1981 | CA | Pacific | HRS | common | spring |
| Yorkstar | CItr14026 | 1968 | NY | Eastern | SWW | common | winter |
| Yorkwin | CItr11855 | 1935 | NY | Eastern | SWW | common | winter |
| Yukon | CItr14583 | 1969 | KS | Great Plains | HRW | common | winter |
| Yumar | PI605388 | 1998 | CO | Great Plains | HRW | common | winter |

^a^ACNO refers to accession number of the varieties.

^b^Year refers to the year of release for varieties developed by breeding programs and collection date for landraces.

Supplemental Table S2. Analysis of molecular variance based on various *a priori* populations of U.S. wheat varieties.

|  | **All varieties** | | | | **Spring varieties** | | | | **Winter varieties** | | | |
| --- | --- | --- | --- | --- | --- | --- | --- | --- | --- | --- | --- | --- |
| **Source of variation** | **df** | **SS** | **VC** | **%** | **df** | **SS** | **VC** | **%** | **df** | **SS** | **VC** | **%** |
| Among habit | 1 | 115,956 | 347 | 8.78 |  |  |  |  |  |  |  |  |
| Within varieties | 751 | 2,705,016 | 3,602 | 91.22 |  |  |  |  |  |  |  |  |
| Among market class | 6 | 313.857 | 512 | 13.25 | 2 | 59511 | 439 | 10.63 | 3 | 137683 | 407 | 11.30 |
| Within varieties | 732 | 2.454.431 | 3,353 | 86.75 | 233 | 859302 | 3688 | 89.37 | 499 | 1595130 | 3197 | 88.70 |
| Among region | 4 | 190,892 | 307 | 8.03 | 4 | 64490 | 294 | 7.38 | 4 | 120841 | 306 | 8.59 |
| Within varieties | 744 | 2,614,067 | 3,514 | 91.97 | 227 | 837641 | 3690 | 92.62 | 512 | 1665363 | 3253 | 91.41 |
| Among hard/soft | 1 | 95.529 | 251 | 6.48 | 1 | 43080 | 400 | 9.64 | 1 | 102222 | 394 | 10.80 |
| Within varieties | 737 | 2,672,759 | 3,627 | 93.52 | 234 | 875733 | 3743 | 90.36 | 501 | 1630890 | 3255 | 89.20 |
| Among red/white | 1 | 53,291 | 190 | 4.91 | 1 | 52732 | 449 | 10.81 | 1 | 31390 | 202 | 5.61 |
| Within varieties | 737 | 2,714,997 | 3,684 | 95.09 | 234 | 866081 | 3701 | 89.19 | 501 | 1701422 | 3396 | 94.39 |
| Among pre-/post-1961 | 1 | 35,495 | 95 | 2.51 | 1 | 39046 | 358 | 8.70 | 1 | 21335 | 77 | 2.18 |
| Within varieties | 751 | 2,785,477 | 3,709 | 97.49 | 234 | 879766 | 3760 | 91.30 | 515 | 1764868 | 3427 | 97.82 |

SS: sum of squares, VC: variance component, %: percentage of variation. All p-values were < .001 and significant at α ≤ .05 after Holm-Bonferroni correction for multiple hypotheses testing.

Supplemental Table S3. Analysis of molecular variance based on regional wheat populations of the U.S.

| **Source of variation** | **df** | **SS** | **VC** | **%** |
| --- | --- | --- | --- | --- |
| **Eastern varieties** |  |  |  |  |
| Among habit | 1 | 7,560 | 712 | 17.54 |
| Within varieties | 203 | 679,785 | 3,349 | 82.46 |
| Among market class | 3 | 32,887 | 389 | 10.73 |
| Within varieties | 194 | 628,071 | 3,238 | 89.27 |
| Among hard/soft | 1 | 11,603 | 299 | 8.28 |
| Within varieties | 196 | 649,355 | 3,313 | 91.72 |
| Among red/white | 1 | 15,409 | 353 | 9.67 |
| Within varieties | 196 | 645,548 | 3,294 | 90.33 |
| Among state | 21 | 124,013 | 324 | 9.52 |
| Within varieties | 183 | 56,332 | 3,078 | 90.48 |
| Among pre-/post-1961 | 1 | 16,426 | 130 | 3.79 |
| Within varieties | 203 | 670,919 | 3,305 | 96.21 |
| **Great Plains varieties** |  |  |  |  |
| Among habit | 1 | 34,665 | 690 | 17.68 |
| Within varieties | 209 | 671,162 | 3,211 | 82.32 |
| Among market class | 6 | 66,777 | 580 | 15.64 |
| Within varieties | 202 | 632,129 | 3,129 | 84.36 |
| Among hard/soft | 1 | 11,627 | 241 | 6.75 |
| Within varieties | 207 | 687,280 | 3,320 | 93.25 |
| Among red/white | 1 | 4,808 | 70 | 2.04 |
| Within varieties | 207 | 694,098 | 3,353 | 97.96 |
| Among state | 5 | 34,473 | 114 | 3.37 |
| Within varieties | 205 | 671,354 | 3,275 | 96.63 |
| Among pre-/post-1961 | 1 | 16,170 | 155 | 4.48 |
| Within varieties | 209 | 689,657 | 3,300 | 95.52 |
| **Northern varieties** |  |  |  |  |
| Among habit | 1 | 28,615 | 532 | 13.35 |
| Within varieties | 106 | 365,831 | 3,451 | 86.65 |
| Among market class | 3 | 38,545 | 627 | 15.57 |
| Within varieties | 100 | 340,137 | 3,401 | 84.43 |
| Among hard/soft | 1 | 3,823 | 38 | 1.02 |
| Within varieties | 102 | 374,856 | 3,675 | 98.98 |
| Among red/white | 1 | 3,878 | 103 | 2.72 |
| Within varieties | 102 | 374,801 | 3,675 | 97.28 |
| Among state | 3 | 28,680 | 229 | 6.10 |
| Within varieties | 104 | 365,766 | 3,517 | 93.90 |
| Among pre-/post-1961 | 1 | 14,313 | 243 | 6.34 |
| Within varieties | 106 | 380,133 | 3,586 | 93.66 |
| **Pacific varieties** |  |  |  |  |
| Among habit | 1 | 4,318 | 134 | 3.39 |
| Within varieties | 58 | 220,479 | 3,801 | 96.61 |
| Among market class | 4 | 46,941 | 897 | 21.70 |
| Within varieties | 55 | 177,856 | 3,234 | 78.30 |
| Among hard/soft | 1 | 20,219 | 611 | 14.77 |
| Within varieties | 58 | 204,578 | 3,527 | 85.23 |
| Among red/white | 1 | 31,036 | 923 | 21.65 |
| Within varieties | 58 | 193,762 | 3,341 | 78.35 |
| Among state | 1 | 6,537 | 166 | 4.23 |
| Within varieties | 58 | 218,260 | 3,763 | 95.77 |
| Among pre-/post-1961 | 1 | 27,403 | 846 | 19.91 |
| Within varieties | 58 | 197,394 | 3,403 | 80.09 |
| **Pacific Northwest varieties** |  |  |  |  |
| Among habit | 1 | 35,906 | 400 | 10.32 |
| Within varieties | 163 | 565,748 | 3,471 | 89.68 |
| Among market class | 6 | 86,269 | 541 | 14.23 |
| Within varieties | 157 | 511,430 | 3,258 | 85.77 |
| Among hard/soft | 1 | 25,075 | 290 | 7.57 |
| Within varieties | 162 | 572,623 | 3,535 | 92.43 |
| Among red/white | 1 | 25,124 | 293 | 7.35 |
| Within varieties | 162 | 572,575 | 3,534 | 92.35 |
| Among state | 3 | 30,902 | 182 | 4.87 |
| Within varieties | 161 | 570,752 | 3,545 | 95.13 |
| Among pre-/post-1961 | 1 | 12,348 | 130 | 3.47 |
| Within varieties | 163 | 589,306 | 3,615 | 96.53 |

SS: sum of squares, VC: variance component, %: percentage of variation.

Supplemental Table S4. Analysis of molecular variance based on wheat market classes.

| **Source of variation** | **df** | **SS** | **VC** | **%** |
| --- | --- | --- | --- | --- |
| **Hard red spring varieties** |  |  |  |  |
| Among region | 4 | 48,017 | 346 | 8.93 |
| Within varieties | 141 | 497,925 | 3,531 | 91.07 |
| Among state | 14 | 102,518 | 426 | 11.18 |
| Within varieties | 131 | 443,424 | 3,385 | 88.82 |
| Among pre-/post-1961 | 1 | 24,171 | 496 | 12.04 |
| Within varieties | 144 | 521,772 | 3,623 | 87.96 |
| **Hard red winter varieties** |  |  |  |  |
| Among region | 3 | 26,091 | 151 | 4.76 |
| Within varieties | 228 | 686,585 | 3,011 | 95.24 |
| Among state | 17 | 93,855 | 224 | 7.17 |
| Within varieties | 214 | 618,820 | 2,892 | 92.83 |
| Among pre-/post-1961 | 1 | 12,566 | 102 | 3.23 |
| Within varieties | 230 | 700,109 | 3,044 | 96.77 |
| **Soft red winter varieties** |  |  |  |  |
| Among region | 4 | 17,684 | 102 | 2.97 |
| Within varieties | 183 | 607,335 | 3,319 | 97.03 |
| Among state | 28 | 138,778 | 311 | 9.22 |
| Within varieties | 159 | 486,240 | 3,058 | 90.78 |
| Among pre-/post-1961 | 1 | 19,265 | 173 | 5.03 |
| Within varieties | 186 | 605,754 | 3,257 | 94.97 |
| **Soft white spring varieties** |  |  |  |  |
| Among region | 2 | 16,545 | 313 | 8.47 |
| Within varieties | 67 | 226,306 | 3,378 | 91.53 |
| Among state | 4 | 31,974 | 387 | 10.65 |
| Within varieties | 65 | 210,878 | 3,244 | 89.35 |
| Among pre-/post-1961 | 1 | 16,560 | 378 | 10.21 |
| Within varieties | 68 | 226,292 | 3,328 | 89.79 |
| **Soft white winter varieties** |  |  |  |  |
| Among region | 2 | 18,725 | 429 | 12.51 |
| Within varieties | 71 | 212,769 | 2,997 | 87.49 |
| Among state | 7 | 38,567 | 328 | 10.10 |
| Within varieties | 66 | 192,927 | 2,923 | 89.90 |
| Among pre-/post-1961 | 1 | 7,907 | 148 | 4.55 |
| Within varieties | 72 | 223,587 | 3,105 | 95.45 |

SS: sum of squares, VC: variance component, %: percentage of variation.

Supplemental Table S5. Genetic diversity (PIC) summaries of U.S. wheat populations.

|  |  | **SNPs** | | | | **Haplotypes** | | | |
| --- | --- | --- | --- | --- | --- | --- | --- | --- | --- |
| **Population** | **No** | **Mean** | **SD** | **Median** | **IQR** | **Mean** | **SD** | **Median** | **IQR** |
| All USA | 753 | .361 | .125 | .396 | .220 | .653 | .176 | .673 | .250 |
| Eastern | 205 | .323 | .156 | .364 | .280 | .615 | .196 | .647 | .262 |
| Great Plains | 211 | .322 | .144 | .346 | .259 | .599 | .190 | .624 | .256 |
| Northern | 108 | .351 | .134 | .384 | .206 | .625 | .176 | .651 | .243 |
| Pacific | 60 | .361 | .140 | .406 | .202 | .608 | .178 | .642 | .242 |
| PNW | 165 | .351 | .135 | .391 | .224 | .630 | .182 | .648 | .249 |
| **Spring by region** |  |  |  |  |  |  |  |  |  |
| All USA | 236 | .375 | .121 | .417 | .178 | .641 | .171 | .668 | .237 |
| Great Plains | 26 | .328 | .151 | .355 | .249 | .559 | .189 | .589 | .246 |
| Northern | 73 | .343 | .146 | .385 | .217 | .598 | .184 | .630 | .239 |
| Pacific | 58 | .359 | .142 | .414 | .216 | .605 | .179 | .639 | .243 |
| PNW | 72 | .356 | .135 | .401 | .191 | .616 | .180 | .647 | .238 |
| **Winter by region** |  |  |  |  |  |  |  |  |  |
| All USA | 517 | .332 | .149 | .376 | .270 | .627 | .191 | .653 | .262 |
| Eastern | 202 | .321 | .157 | .362 | .278 | .613 | .197 | .648 | .260 |
| Great Plains | 185 | .303 | .154 | .313 | .289 | .579 | .198 | .607 | .269 |
| Northern | 35 | .290 | .159 | .320 | .294 | .555 | .206 | .586 | .294 |
| PNW | 93 | .310 | .164 | .350 | .312 | .585 | .200 | .612 | .270 |
| **Market classes** |  |  |  |  |  |  |  |  |  |
| HRS | 150 | .361 | .134 | .403 | .200 | .616 | .177 | .646 | .235 |
| HWS | 16 | .319 | .158 | .375 | .250 | .561 | .194 | .602 | .242 |
| SWS | 70 | .334 | .152 | .382 | .243 | .588 | .190 | .624 | .251 |
| HRW | 232 | .296 | .154 | .307 | .289 | .570 | .200 | .598 | .281 |
| HWW | 9 | .277 | .177 | .346 | .247 | .516 | .207 | .568 | .296 |
| SRW | 188 | .320 | .159 | .359 | .280 | .611 | .197 | .646 | .259 |
| SWW | 74 | .301 | .161 | .339 | .285 | .568 | .202 | .606 | .275 |

No: number of varieties; SD: standard deviation; IQR: interquartile range.

Supplemental Table S6. Number of haplotype alleles observed in U.S. wheat varieties from different periods.

| **By decade** | | | | | | **By equal number of varieties** | | | | | |
| --- | --- | --- | --- | --- | --- | --- | --- | --- | --- | --- | --- |
| **Period** | **Varieties** | **Total** | **% Lost** | **% New** | **% Total** | **Period** | **Varieties** | **Total** | **% Lost** | **% New** | **% Total** |
| **Both spring and winter** | | | | | | | | | | | |
| pre-1930 | 90 | 12,710 | 0 | 0 | 100 | until 1928 | 84 | 12,383 | 0 | 0 | 100 |
| 1930-39 | 49 | 10,694 | 28 | 13 | 84 | 1928-44 | 84 | 12,302 | 21 | 20 | 99 |
| 1940-49 | 56 | 10,659 | 29 | 13 | 84 | 1944-61 | 84 | 11,904 | 24 | 20 | 96 |
| 1950-59 | 45 | 10,212 | 33 | 13 | 80 | 1961-72 | 84 | 11,695 | 27 | 22 | 94 |
| 1960-69 | 72 | 11,297 | 30 | 18 | 89 | 1972-77 | 84 | 11,417 | 30 | 23 | 92 |
| 1970-79 | 143 | 13,091 | 26 | 29 | 103 | 1977-82 | 84 | 11,773 | 29 | 24 | 95 |
| 1980-89 | 165 | 13,982 | 25 | 35 | 110 | 1982-88 | 84 | 12,106 | 29 | 27 | 98 |
| 1990-99 | 92 | 12,874 | 27 | 28 | 101 | 1988-93 | 84 | 12,367 | 28 | 27 | 100 |
| 2000-15 | 41 | 9,870 | 39 | 16 | 78 | 1993-2014 | 81 | 11,857 | 30 | 26 | 96 |
| **Spring varieties** | | | | | | | | | | | |
| pre-1930 | 25 | 8,593 | 0 | 0 | 100 | until 1930 | 27 | 8,810 | 0 | 0 | 100 |
| 1930-39 | 18 | 6,980 | 31 | 13 | 81 | 1930-44 | 27 | 7,517 | 28 | 13 | 85 |
| 1940-49 | 18 | 6,277 | 38 | 11 | 73 | 1944-66 | 27 | 8,035 | 31 | 23 | 91 |
| 1950-59 | 9 | 5,472 | 48 | 11 | 64 | 1966-73 | 27 | 7,370 | 38 | 22 | 84 |
| 1960-69 | 22 | 7,431 | 36 | 22 | 86 | 1973-78 | 27 | 7,632 | 37 | 24 | 87 |
| 1970-79 | 50 | 8,733 | 31 | 33 | 102 | 1978-83 | 27 | 7,473 | 40 | 25 | 85 |
| 1980-89 | 59 | 9,461 | 29 | 39 | 110 | 1983-87 | 27 | 7,894 | 37 | 26 | 90 |
| 1990-99 | 24 | 8,231 | 34 | 30 | 96 | 1987-94 | 27 | 8,184 | 35 | 28 | 93 |
| 2000-15 | 11 | 5,339 | 53 | 15 | 62 | 1994-2011 | 20 | 6,830 | 44 | 22 | 78 |
| **Winter varieties** | | | | | | | | | | | |
| pre-1930 | 65 | 11,076 | 0 | 0 | 100 | until 1928 | 58 | 10,548 | 0 | 0 | 100 |
| 1930-39 | 31 | 8,938 | 32 | 12 | 81 | 1928-46 | 58 | 10,996 | 21 | 25 | 104 |
| 1940-49 | 38 | 9,523 | 30 | 16 | 86 | 1946-60 | 58 | 10,418 | 23 | 22 | 99 |
| 1950-59 | 36 | 8,850 | 33 | 13 | 80 | 1960-72 | 58 | 10,101 | 27 | 22 | 96 |
| 1960-69 | 50 | 9,655 | 30 | 18 | 87 | 1972-77 | 58 | 9,601 | 31 | 22 | 91 |
| 1970-79 | 93 | 11,233 | 26 | 27 | 101 | 1977-83 | 58 | 10,100 | 28 | 24 | 96 |
| 1980-89 | 106 | 12,052 | 25 | 33 | 109 | 1983-88 | 58 | 10,675 | 27 | 29 | 101 |
| 1990-99 | 68 | 11,144 | 27 | 28 | 101 | 1988-94 | 58 | 10,729 | 27 | 28 | 102 |
| 2000-15 | 30 | 8,594 | 39 | 16 | 78 | 1994-2014 | 53 | 10,020 | 30 | 25 | 95 |

Total refers to the number of alleles counted in varieties from a given period for the 1585 haplotype loci. Percentage lost, new, and total alleles are proportion of the number of alleles in the earliest subpopulation (indicated in red) for both spring and winter, spring, and winter varieties.
